# Inhibition of NF-κB in astrocytes is sufficient to delay neurodegeneration induced by proteotoxicity in neurons

**DOI:** 10.1186/s12974-018-1278-2

**Published:** 2018-09-11

**Authors:** Y. X. Li, O. C. M. Sibon, P. F. Dijkers

**Affiliations:** 1 Department of Cell Biology, University of Groningen, Antonius Deusinglaan 1, 9713AV Groningen, The Netherlands; 2 Hubrecht Institute for Developmental Biology and Stem Cell Research, Royal Netherlands Academy of Arts and Sciences (KNAW) and UMC Utrecht, Utrecht, The Netherlands

**Keywords:** Neurodegeneration, Astrocytes, NF-κB, Cell-non-autonomous, *Drosophila*, Polyglutamine diseases, Spinocerebellar ataxia, Neuroimmunology

## Abstract

**Background:**

Most neurodegenerative diseases associated with protein aggregation are hallmarked by activation of astrocytes. However, how astrocytes are activated or which signaling pathways in astrocytes contribute to pathogenesis is not clear. One long-standing question is whether the responses in astrocytes are due to stress or damage in astrocytes themselves, or because of astrocytic responses to cellular stress or damage in neurons. Here, we examine responses in astrocytes induced by expression of disease-associated, aggregation-prone proteins in other cells. We also examine the consequences of these responses in astrocytes in a model for neurodegeneration.

**Methods:**

We first examined a role for intracellular astrocytic responses in a *Drosophila* model for Spinocerebellar ataxia type 3 (SCA3, also known as Machado–Joseph disease), a disease caused by expansion of the polyglutamine (polyQ) stretch in the *ATXN3* gene. In this *Drosophila* SCA3 model, eye-specific expression of a biologically relevant portion of the *ATXN3* gene, containing expanded polyQ repeats (SCA3^polyQ78^) was expressed. In a candidate RNAi screen in the *Drosophila* SCA3 model, we analyzed whether downregulation of expression of specific genes in astrocytes affected degeneration induced by SCA3^polyQ78^ expression in *Drosophila* eyes. We next examined the role of astrocytes in response to proteotoxic stress in neurons induced by SCA3^polyQ78^ expression or amyloid beta peptides, associated with Alzheimer’s disease.

**Results:**

Eye-specific expression of SCA3^polyQ78^ resulted in the presence of astrocytes in the eye, suggesting putative involvement of astrocytes in SCA3. In a candidate RNAi screen, we identified genes in astrocytes that can enhance or suppress SCA3^polyQ78^-induced eye degeneration. Relish, a conserved NF-κB transcription factor, was identified as an enhancer of degeneration. Activity of Relish was upregulated in our SCA3 model. Relish can exert its effect via Relish-specific AMPs, since downregulation of these AMPs attenuated degeneration. We next examined Relish signaling in astrocytes on neurodegeneration. Selective inhibition of Relish expression specifically in astrocytes extended lifespan of flies that expressed SCA3^polyQ78^ exclusively in neurons. Inhibition of Relish signaling in astrocytes also extended lifespan in a *Drosophila* model for Alzheimer’s disease.

**Conclusions:**

Our data demonstrate that astrocytes respond to proteotoxic stress in neurons, and that these astrocytic responses are important contributors to neurodegeneration. Furthermore, our data demonstrate that activation of NF-κB transcription factor Relish in astrocytes, induced by proteotoxic stress in neurons, enhances neurodegeneration, and that specific Relish inhibition in astrocytes extends lifespan. Our data provide direct evidence for cell-non-autonomous contributions of astrocytes to neurodegeneration, with possible implications for therapeutic interventions in multiple neurodegenerative diseases.

**Electronic supplementary material:**

The online version of this article (10.1186/s12974-018-1278-2) contains supplementary material, which is available to authorized users.

## Background

A hallmark of many age-related neurodegenerative diseases is the presence of protein aggregates, which precedes the onset of clinical symptoms. Genetic profiling of transcriptional changes in neurodegenerative diseases resulted in the identification of glial genes whose altered expression could potentially contribute to pathogenesis [1, 2]. Besides this, another characteristic in most, if not all neurodegenerative diseases associated with protein aggregation is the activation of astrocytes [3]. Recently, it was demonstrated that a subtype of astrocytes is neurotoxic and is present in most neurodegenerative diseases [4].

The presence of aggregates in astrocytes can result in their activation and subsequent signaling to neurons [5]; reviewed in [6]. However, it is unclear whether signals from aggregate-expressing neurons can modulate the activity of astrocytes. Furthermore, the putative consequences of signaling from astrocytes that are activated by neuronal proteotoxic stress on the functioning of neurons are not known.

Astrocytes are involved in essential CNS functions, including metabolic functions, regulating levels of neurotransmitters, maintaining the blood-brain barrier, as well as immune defense, thus contributing to neuronal homeostasis [7]. Astrocytes are activated early in neurodegenerative diseases at times that may precede the appearance of aggregates [6]. This activation only occurs in specific areas of the brain, i.e., those that are primarily affected by the respective diseases, such as for example the striatum in Huntington’s disease (HD), the pons in SCA3, and the cortex in Alzheimer’s disease (AD) [8, 9]. Alterations in function of astrocytes can contribute to pathogenesis in a mouse model for HD, and therefore astrocytes have been suggested as potential therapeutic targets [10]. In this model, the HD-associated, aggregation-prone protein was amongst others expressed in the brain, including neurons, microglia, and astrocytes. Thus, in this model, cell-autonomous activation of astrocytes (in response to proteotoxic stress) as well as cell-non-autonomous activation of astrocytes (in response to proteotoxic stress in neurons) could contribute to pathogenesis. There is ample evidence of cell-autonomous activation of astrocytes in the pathogenesis of neurodegenerative diseases (reviewed in [6, 11]). However, how signaling from aggregate-expressing neurons can influence signaling in astrocytes and how responses from astrocytes can modulate neurodegeneration is unknown.

We investigated if and how astrocytes contribute to neurodegenerative diseases in a cell-non-autonomous manner, within the context of an intact animal. Examining astrocytes in an in vivo model is key, given that their morphology and activity change when taken outside their physiological context (reviewed in [12]). For this, we conducted a dedicated RNAi screen to selectively knock down individual genes in astrocytes in a *Drosophila melanogaster* model of the polyQ disease SCA3. In SCA3, the *ATXN3* gene contains an expanded CAG repeat (coding for glutamine) that leads to the expression of a misfolded, aggregation-prone ATXN3 polyglutamine protein. These misfolded polyQ-containing proteins accumulate intracellularly, resulting in neuronal damage and activation of astrocytes [13]. In the brains of patients as well as of animal models, proteolytic cleavage of a fragment of the gene containing the polyQ-containing stretch can mediate the toxicity of expanded polyQ [14–16]. We expressed this biologically relevant, truncated part of the *ATXN3* gene containing an expanded polyQ stretch (SCA3^polyQ78^) exclusively in *Drosophila* eyes and simultaneously downregulated expression (by RNAi) of candidate genes exclusively in astrocytes. This setup would provide insight into the putative cell-non-autonomous contributions of astrocytes to neurodegeneration induced by proteotoxicity in neurons. In a candidate RNAi screen, we identified genes in astrocytes that cell-non-autonomously influenced degeneration. This demonstrates the relevance of cell-non-autonomous signaling in astrocytes in neurodegeneration. We further explored the contribution of NF-κB transcription factor *Relish*, which was identified as an enhancer in our screen, and its activity was upregulated in our SCA3 model. We investigated the role of *Relish* in astrocytes in SCA3, as well as in a model for Alzheimer’s disease.

## Methods

### *Drosophila* strains

All fly lines were maintained at 25 °C on standard fly food unless indicated otherwise. The following stocks were obtained from the Bloomington Drosophila stock center (BDSC, Bloomington, Indiana, U.S.A.): *UAS-MJD.tr-Q27* (8149), *UAS-MJD.tr-Q78* (8150), *QUAS-mCD8-GFP* (30003), *UAS myr-RFP* (7118), two *tub-QS* lines (52112 and 30024), *alrm-Gal4* (67031); *GMR-Gal4* (1104); *daughterless-Gal4* (8641); *UAS-Relish RNAi #2* (33661); Relish E20 mutant (9457). The following stocks were from Vienna Drosophila Research Center (VDRC): *UAS-Relish RNAi #1* (49414-GD), *UAS-Attacin A RNAi* (50320-GD), *UAS-Cecropin A* (42859-GD). The stocks used in the screen are all from VDRC. *UAS-GFP-Relish* has been described [17]. *GMR-QF2* (BDSC #59283) *nSyb-QF2* (BDSC 51956), the *CyO-tub-QS* balancer as well as the pQUAST vector were a gift from C. Potter (Baltimore, MD, USA). *UAS-necrotic-Abeta42* flies were a gift from D. Crowther (Cambridge University, Cambridge, United Kingdom) and have been described previously [18]. All flies were backcrossed to w1118 flies. We generated *QUAST-SCA3*^*polyQ27*^ and *QUAST-SCA3*^*polyQ78*^ transgenic flies by amplifying the corresponding cDNA from gDNA of stock #8149 (UAS-*SCA3*^*polyQ27*^) or #8150 (UAS-*SCA3*^*polyQ78*^), using the following UAS-specific primers: 5′- ATAGGGAATTGGGAATTCGTT-3′ and 5′- CAATTATGTCACACCACAGAA-3′ and cloned into pUAST using EcoRI and XbaI. We generated pQUAST-necrotic-Aβ42 by amplifying from gDNA from *UAS-necrotic-Abeta42* flies using 5′-cgaattcaacATGgcgagcaaagtctcgatc-3′ and 5’-ctctagaTTACGCAATCACCACGCCGC-3′ and cloned into pQUAST using EcoRI and XbaI. Constructs were verified by sequencing. Transgenic fly lines were generated in the w1118 background, using BestGene (Chino Hills, CA, USA).

### Genetics

To independently and simultaneously manipulate gene expression in either the eyes or neurons, or astrocytes, we used the QUAS-QF system to express constructs in neurons or in the eyes and UAS-Gal4 to manipulate gene expression in astrocytes (using *alrm-Gal4*). We suppressed QF-dependent expression by expressing QS under control of the tubulin promoter. A schematic drawing of the genetic systems involved is shown in Additional files 1a and 7b. To analyze astrocyte-associated genes in *SCA3*^*polyQ78*^-induced eye degeneration, we used the following fly line: *GMR-QF2/(Y); QUAS-SCA3*^*polyQ78*^*:: alrm-Gal4/CyO-tub-QS*. In this line, expression of *SCA3*^*polyQ78*^ is suppressed by QS, which inhibits QF2. To analyze genes in astrocytes on the degenerative SCA3 eye phenotype, we crossed this line to different UAS constructs. To quantify eye degeneration by analyzing levels of mCD8-GFP, we used the line *GMR-QF2/(Y)*; *QUAS-SCA3*^*polyQ78*^*/CyO-tub-QS: alrm-Gal4: QUAS-mCD8-GFP*, and crossed them to UAS lines or w1118 flies. The data depicted are all from female flies, although male flies yielded similar results. To analyze the effect of *SCA3*^*polyQ78*^ in neurons and a possible effect of astrocytes on the SCA3^*polyQ78*^-induced pathogenesis, we used the following line: *tub-QS/(y)*; *QUAS-SCA3*^*polyQ78*^*:: alrm-Gal4*; *nSyb-QF2: tub-QS*. We used two copies of *tub-QS*, since one copy was not able to suppress the expression of *SCA3*^*polyQ78*^. We supplemented adult flies with quinic acid to suppress QS and allow expression of *SCA3*^*polyQ78*^ only in adulthood. Data of females are depicted, although data in males yielded similar results.

### Analysis of eye degeneration

For each line we analyzed, we calculated the fraction of the eyes that showed a SCA3^polyQ78^-induced degenerative phenotype (necrotic spots) as shown previously [19] of at least 40 flies. The data of eye degeneration that are shown are all of female flies, although male flies yielded similar results. In all the figures, the percentage of eye degeneration is calculated as the fraction of the eyes containing necrotic spots. The results were average of at least three independent experiments −/+SEM. *p < 0.05; **p < 0.01.

### Real-time quantification PCR (RT-qPCR)

We collected 120 fly heads per point to isolate total RNA using the RNeasy Mini Kit (Qiagen). To collect the heads, flies were frozen in liquid nitrogen and decapitated by vortexing. Fly heads were collected using a mesh. M-MLV reverse transcriptase (Invitrogen, 28025-013) was used to transcribe RNA into cDNA. Relative quantification of the gene expression level was determined in CFX ConnectTM (Bio-Rad) by using iQTM SYBR® Green Supermix (Bio-Rad Laboratories, Inc.). For all the samples, gene expression levels were normalized to a housekeeping gene, *ribosomal protein 49* (*RP49*). Analysis of knockdown of gene expression was determined in adult flies.

Primer pairs used for QPCR:

- *RP49*: 5′-CCGCTTCAAGGGACAGTATC-3′/5′-GACAATCTCCTTGCGCTTCT-3′
- *IM1*: 5′-TGCCCAGTGCACTCAGTATC-3′/5′-GATCACATTTCCTGGATCGG-3′
- *IM2*: 5′-AAATACTGCAATGTGCACGG-3′/5′-ATGGTGCTTTGGATTTGAGG-3′
- AttA: 5′-ACAAGCATCCTAATCGTGGC-3′/5′-GGTCAGATCCAAACGAGCAT-3′
- *AttC*: 5′-CCAATGGCTTCAAGTTCGAT-3′/5′-AGGGTCCACTTGTCCACTTG-3′
- *DptA*: 5′-ACCGCAGTACCCACTCAATC-3′/5′-ACTTTCCAGCTCGGTTCTGA-3′
- *CecA*: 5′-GAACTTCTACAACATCTTCGT-3′/5′-TCCCAGTCCCTGGATTGT-3′
- Relish: 5′-AGCAGTGGCGCACTAAAGTT-3′/5′-GATGGCTGACCATTCGTTTT-3′

Results are average of at least three independent experiments −/+SEM. *p < 0.05; **p < 0.01; ***p < 0.001.

### Western blotting

For examining HA-tagged SCA3^polyQ27^ or SCA3^polyQ78^ levels and aggregation and their effect on eye viability (using CD8-GFP), 2-day-old flies were used. At least 30 fly heads per genotype were collected, lysed in Laemli buffer by sonification, and boiled at 95 °C for 5 min. Samples were separated on 12.5% SDS-PAGE gels and transferred to PVDF membranes (Millipore, Fisher Scientific). After blocking in 5% (*w*/*v*) non-fat dried milk in PBST for 1 h, the membrane was incubated with primary antibody overnight at 4 °C. The following are antibodies were used: rat anti-HA-Peroxidase (Roche Diagnostics GmbH, Germany), rabbit anti-GFP (A11122; Invitrogen) mouse anti-RFP (6G6 ChromoTek) 6E10 Ab was purchased from Covenance and mouse anti-alpha tubulin from Sigma (T5138). After incubation with secondary antibody, ECL (Amersham) signal was detected in a ChemiDocTM Touch (Bio-Rad). The intensity of the bands was analyzed by using ImageJ (National Institutes of Health, USA). Quantification of western blots was of at least three independent experiments unless indicated otherwise, *p < 0.05; **p < 0.01.

### Lifespans

To determine the lifespan of flies, a maximum of 20 flies was kept in a vial. At least 80 flies per condition were analyzed. Every 2 days flies were transferred into new vials containing fresh food. Data are representative of female flies, although similar results were obtained with males. The following fly lines were used to determine the effect of genes in astrocytes on survival: (1) *tub-QS/(y)*; *QUAS-SCA3*^*polyQ78*^*:: alrm-Gal4/CyO; nSyb-QF2:: tub-QS/TM6B* (*SCA3*^*polyQ78*^ line), (2) *tub-QS/(y)*; *alrm-Gal4/CyO*; *nSyb-QF2*: *tub-QS/TM6B* (control line for *SCA3*^*polyQ78*^), (3) *QUAS-necrotic-Abeta42:: alrm-Gal4/CyO-QS*; *nSyb-QF2* (Abeta42 line), and (4) *alrm-Gal4/CyO-QS*; *nSyb-QF2* (control line for *Abeta42*). These lines were used to test the effect of astrocyte-specific genes on flies that express *SCA3*^*polyQ78*^ in neurons in adulthood or Abeta42 peptides. Expression of *SCA3*^*polyQ78*^ was induced in neurons of adult flies by inhibiting QS through supplementing the food with quinic acid. Quinic acid was used in a concentration of 250 mg/ml with fixed pH of 7. 250 μl of quinic acid solution was put on top of the food to cover the entire surface and used when the solution was completely absorbed into the food [20]. Lifespan curves were analyzed in GraphPad Prism (GraphPad Software, San Diego, CA, USA) and statistical significance was analyzed by Log-rank (Mantel-Cox) test.

### Climbing assay

For each cross, 5 vials of flies, containing a maximum of 20 flies per vial, were analyzed. Here, we analyzed males, as the climbing ability in females varies more between the individuals of a group. To determine the mobility of flies, we analyzed their climbing ability. For this, the vial was divided into four compartments, with the lowest compartment numbered with 1 (slow climbers) and the highest with number 4 (fast climbers). Flies were tapped down to the bottom of vials and were allowed 10 s to climb up, after which a picture was taken. The numbers of flies in each compartment were counted for each time point. To get better separation of the differences, flies in compartment 1 got a score of 1; flies in compartment 2 got a score of 3; flies in compartment 3 got a score of 5, and flies in compartment 4 got a score of 7. Subsequently, the average score of each time point was determined by dividing the total score by the total number of flies. The climbing test was repeated three times for each group, and the average of the three times was shown.

### Drosophila brain and microscopy

Heads of *Drosophila* females were collected in ice-cold PBS, fixed in 3.7% formaldehyde solution (0.1% triton x-100 in PBS) for 15 min, and washed five times in PBS. Next, *Drosophila* brains were dissected in PBS and stained with DAPI solution (0.2 mg/ml, 2% BSA and 0.1% Triton x-100 in PBS) at room temperature. After washing with PBS for three times, the brains were mounted in 80% glycerol on slides and imaged by a Leica SP8 confocal microscope.

## Results

**Fig. 1:**
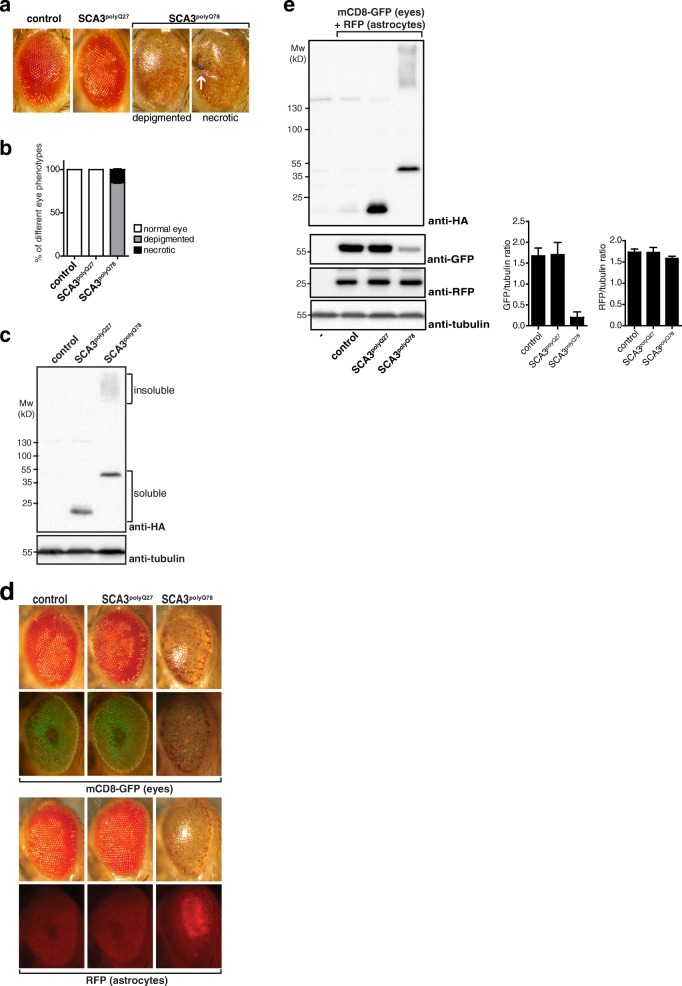
Analysis and quantification of the effects induced by expression of *SCA3*^*polyQ78*^. **a** Representative pictures of a control eye, and eyes expressing *SCA3*^*polyQ27*^ or *SCA3*^*polyQ78*^. Expression of *SCA3*^*polyQ78*^ but not *SCA3*^*polyQ27*^ in *Drosophila* eyes resulted in loss of pigment (“depigmented”), as well as the presence of necrotic patches (“necrotic”, indicated with arrow). **b** Quantification of the fraction of the eyes as shown in **a** that have a normal appearance, display a loss of pigment or contain necrotic patches; the fraction of *SCA3*^*polyQ78*^ eyes containing necrotic patches was used as a quantitative measure for degeneration. Data are representative of at least three independent experiments. SEM = 0.863. **c** Western blot analysis of fly head lysates expressing eye-specific, HA-tagged SCA3^polyQ27^, or SCA3^polyQ78^*.* A fraction of SCA3^polyQ78^ but not SCA3^polyQ27^ was SDS-insoluble. Tubulin was used as a control for equal loading. **d** Expression of mCD8-GFP in the eye to analyze SCA3^polyQ78^-induced eye degeneration and analysis of SCA3^polyQ78^ expression on the localization of astrocytes (expressing RFP). Top panel: GFP fluorescence of a representative control eye or eyes coexpressing SCA3^polyQ27^ or SCA3^polyQ78^. Bottom panel: the localization of myr-RFP-expressing (RFP) astrocytes was analyzed in control eyes or eyes expressing SCA3^polyQ27^, or SCA3^polyQ78^. **e** Lysates of control flies or flies expressing mCD8-GFP in the eyes and myr-RFP in astrocytes together in the absence or presence of SCA3^polyQ27^ or SCA3^polyQ78^ were analyzed for expression of HA-tagged SCA3, GFP, and RFP. Tubulin was used as an equal loading control. Genotypes in **a**–**c**: control, *GMR-QF2/+.* SCA3^polyQ27^, *GMR-QF2/+*; *QUAS-SCA3*^*polyQ27*^*/+.* SCA3^polyQ78^, *GMR-QF2/+*; *QUAS-SCA3*^*polyQ78*^*/+.* Genotypes in **d**, top panel: control, *GMR-QF2/+*; *QUAS-mCD8-GFP/+.* SCA3^polyQ27^, *GMR-QF2/+*; *QUAS-SCA3*^*polyQ27*^*/+*; *QUAS-mCD8-GFP/+.* SCA3^polyQ78^: *GMR-QF2/+*; *QUAS-SCA3*^*polyQ78*^*/+*; *QUAS-mCD8-GFP/+.* Bottom: control, *GMR-QF2/+*; *alrm-Gal4::UAS-myr-RFP/+.* SCA3^polyQ27^, *GMR-QF2/+*; *alrm-Gal4::UAS-myr-RFP/QUAS-SCA3*^*polyQ27*^*.* SCA3^polyQ78^: *GMR-QF2/+*; *alrm-Gal4::UAS-myr-RFP/QUAS-SCA3*^*polyQ78*^*.* Genotypes in **e**: as in **d** except that, where indicated, both *QUAS-mCD8-GFP* (eyes) or *alrm-Gal4::UAS-myr-RFP* (astrocytes) were expressed

Expression of a biologically relevant, truncated form of the human *ATXN3* gene containing an expansion of the glutamine region (trSCA3 Q78, containing 78 glutamines, hereafter referred to as *SCA3*^*polyQ78*^) in *Drosophila* eyes resulted in progressive degeneration of photoreceptor cells [21], accompanied by eye depigmentation as well as depigmentation accompanied by black necrotic spots (depigmented and necrotic, Fig. 1a; arrow indicates necrotic spots). No such effects were seen in control eyes or eyes expressing *SCA3*^*polyQ27*^, containing a non-pathogenic length of glutamines (*SCA3*^*polyQ27*^; Fig. 1a) [21]. Quantification of the fraction of the eyes with a normal appearance, displaying depigmentation or necrotic spots is shown in Fig. 1b. Only expression of *SCA3*^*polyQ78*^ resulted in depigmentation or necrotic spots. We quantified the extent of degeneration by determining the fraction of the eyes containing necrotic spots. This quantification provides a quick and reproducible means of analysis. To see whether SCA3^polyQ78^-induced degeneration was accompanied by decreased solubility of SCA3^polyQ78^, lysates of fly heads were analyzed. Expression of SCA3^polyQ78^ but not SCA3^polyQ27^ resulted in the presence of high molecular weight insoluble proteins (Fig. 1c), which have previously been identified as aggregates using immunofluorescence [22].

We used another robust, sensitive way to quantify degeneration, by coexpressing membrane-targeted mCD8-GFP [23] with *SCA3*^*polyQ27*^ or S*CA3*^*polyQ78*^ in the eyes. Expression of *SCA3*^*polyQ78*^ but not *SCA3*^*polyQ27*^ resulted in a decrease of mCD8-GFP levels, indicative of degeneration (Fig. 1d, top panel). Analysis of mCD8-GFP expression levels in *SCA3*^*polyQ78*^*-*expressing eyes in lysates of fly heads allows for quantification of degeneration (Fig. 1e). Both the fraction of necrotic eyes and GFP levels were used as readouts to determine the effect of downregulation of specific genes in astrocytes on degeneration.

To analyze a putative cell-non-autonomous role for astrocytes in *SCA3*^*polyQ78*^-induced eye degeneration, we used two binary expression systems that act independently [20], UAS-Gal4 and QUAS-QF2 (Additional file 1a). Indeed, expression of fluorescent proteins in astrocytes (RFP) or the eyes (GFP) shows specific non-overlapping expression in the respective tissues (Additional file 1b). We analyzed whether the location of astrocytes (expressing RFP) was altered upon expression of *SCA3*^*polyQ78*^ in the eyes. We observed the presence of astrocytes in *SCA3*^*polyQ78*^-expressing eyes but not in control eyes or eyes expressing *SCA3*^*polyQ27*^ (Fig. 1d, bottom panel). The response in *SCA3*^*polyQ78*^-expressing flies is quite robust, as they all displayed expression of RFP in the eyes (Additional file 2). The number of astrocytes in all samples in Fig. 1d is comparable, as total RFP levels in fly heads were similar (Fig. 1e). Thus, the presence of astrocytes in *SCA3*^*polyQ78*^-expressing eyes suggests a response in astrocytes, which warrants further investigation into a putative cell-non-autonomous role of astrocytes in degeneration.

Our *Drosophila* SCA3 model allows specific analysis of cell-non-autonomous contributions of astrocytes to the degenerative SCA3 eye phenotype. We selected genes (many of them evolutionarily conserved; Additional file 3) potentially involved in (1) communication between neurons and astrocytes, such as neuropeptides and their cognate receptors (2) immune signaling pathways, such as nuclear factor kappa B (NF-κB) and (3) potential receptors for cytokines, neuropeptides, aggregates, or damage-associated molecular patterns. Some of the genes that were selected have either been implicated in cell-autonomous activation of astrocytes [6] or are upregulated in the degenerative fly brain [24, 25]. However, the role of these genes in cell-non-autonomous activation of astrocytes in response to proteotoxic stress in neurons and subsequent contribution to neurodegeneration still remained to be determined. In a candidate RNAi screen, RNAi constructs targeting a set of 156 selected genes were expressed exclusively in astrocytes in flies expressing *SCA3*^*polyQ78*^ exclusively in the eyes, and the extent of eye degeneration was analyzed by determining the fraction of necrotic spots. The screen revealed both suppressors (*n* = 26; RNAi against these genes enhanced degeneration) as well as enhancers (*n* = 20; RNAi against these genes reduces degeneration) of the SCA3 eye phenotype (Additional file 3). These results underscore the relevance of astrocytes in the progression of *SCA3*^*polyQ78*^-induced eye degeneration.

**Fig. 2:**
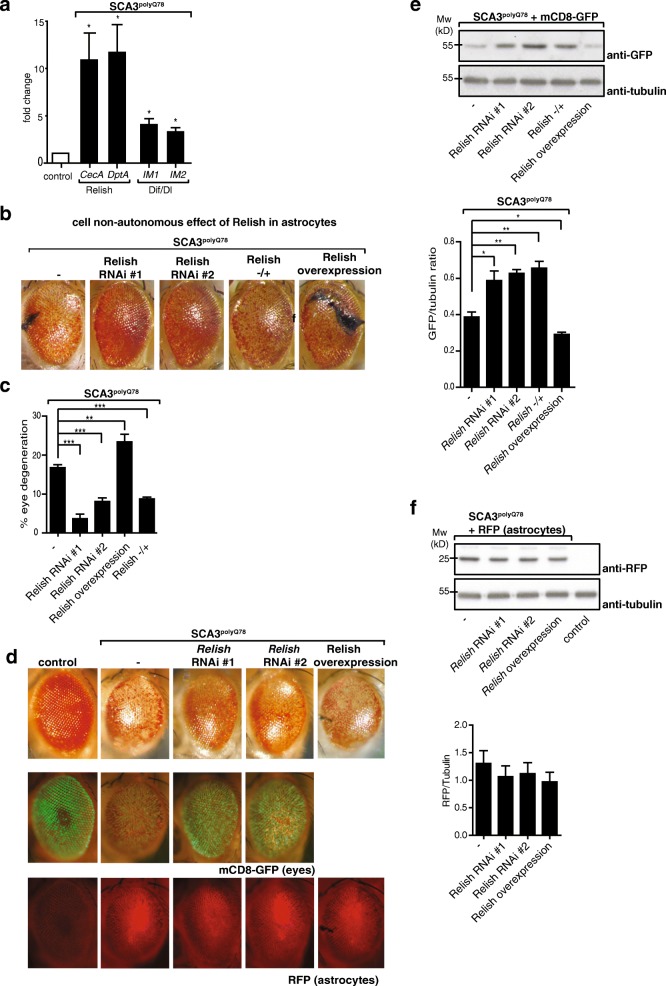
Effect of NF-κB transcription factor Relish in astrocytes on *SCA3*^*polyQ78*^-induced degeneration. **a** Quantification of Relish or Dif/Dorsal activation in the head upon eye-specific expression of *SCA3*^*polyQ78*^. Heads of control flies or flies expressing *SCA3*^*polyQ78*^ in the eyes were analyzed for expression of Relish target genes (*CecA*, *DptA*) or Dif/Dorsal target genes (*IM-1*, *IM-2*). **b** Effect of modulation of Relish expression in astrocytes on *SCA3*^*polyQ78*^-induced eye degeneration. Representative images of fly eyes expressing *SCA3*^*polyQ78*^ (−) or *SCA3*^*polyQ78*^ together with either astrocyte-specific expression of two independent RNAi constructs targeting *Relish* (*Relish* RNAi #1 and *Relish* RNAi #2), a *Relish* overexpression construct (Relish overexpression) or flies heterozygous for *Relish* (Relish −/+). **c** Quantification of the fraction of degeneration of the eyes shown in **b**. In these and other eye quantification experiments, at least 80 eyes of females were counted per genotype. Data are representative of at least three independent experiments ± SEM. **d** The effect of modulation of *Relish* expression in astrocytes on GFP fluorescence in *SCA3*^*polyQ78*^-expressing eyes together with eye-specific mCD8-GFP and on the localization of astrocytes (expressing myr-RFP). Representative images of control eyes, eyes expressing mCD8-GFP, and astrocyte-specific myr-RFP (RFP) or together with coexpression of *SCA3*^*polyQ78*^ in the absence or presence of *Relish* RNAi constructs (*Relish* RNAi #1 and *Relish* RNAi #2) or *Relish* overexpression in astrocytes. **e** Quantification of the effect of Relish signaling in astrocytes on SCA3^polyQ78^-induced degeneration by using eye-specific expression of mCD8-GFP. Levels of mCD8-GFP were determined in lysates of fly heads of flies expressing *SCA3*^*polyQ78*^ together with mCD8-GFP in the eyes and the effect of astrocyte-specific expression of *Relish* RNAi constructs (*Relish* RNAi #1 and *Relish* RNAi #2) in astrocytes, heterozygosity for *Relish* (*Relish −/+*), or overexpression of *Relish* in astrocytes was analyzed. Tubulin was used as a control for equal loading. Quantification of western blots is of at least three independent experiments. **f** Analysis of myr-RFP expression in astrocytes in lysates of control flies or flies expressing myr-RFP in astrocytes together with eye-specific SCA3^polyQ78^ in the presence or absence of astrocyte-targeted *Relish* RNAi (*Relish* RNAi #1 or #2) or *Relish* overexpression. Quantification of western blots in two independent experiments. Genotypes: **a** control, *GMR-QF2/+*. SCA3^polyQ78^, *GMR-QF2/+*; *QUAS- SCA3*^*polyQ78*^*/+.* **b**, **c** -, *GMR*-*QF2/+*; *QUAS-SCA3*^*polyQ78*^*:: alrm-Gal4/+. Relish* RNAi #1, *GMR*-QF2/+; *QUAS-SCA3*^*polyQ78*^*:: alrm*-Gal4/*UAS-Relish RNAi #1*. *Relish* RNAi #2, *GMR-QF2/+*; *QUAS-SCA3*^*polyQ78*^*:: al*rm*-Gal4/UAS-Relish RNAi #2*. Relish overexpression, *GMR-QF2/+*; *QUAS-SCA3*^*polyQ78*^*:: alrm*-Gal4*/UAS-GFP-Relish*; *Relish−/+*, *GMR-QF2/+*; *QUAS-SCA3*^*polyQ78*^*:: alrm*-Gal4; *Relish E20/+.* **d**–**f** As in **b**, but with additional eye-specific expression of *QUAS-CD8-GFP* or astrocyte-specific expression of *UAS-myr-RFP*

One of the genes identified in the screen was NF-κB transcription factor *Relish* (orthologous to mammalian *NF-κB1*). Downregulation of *Relish* expression by RNAi, but also downregulation of several other genes in the Relish pathway, suppressed the SCA3 phenotype. While NF-κB has been implicated in activation of astrocytes in neurodegeneration (reviewed in [6, 26]), a cell-non-autonomous role of NF-κB activation in astrocytes through signals from neurons and subsequent effect on neurodegeneration remains to be determined. In *Drosophila*, there are two independent NF-κB pathways, activating transcription factors Relish or Dif/Dorsal respectively. We examined expression of anti-microbial peptides (AMPs) in our SCA3 model, transcriptional NF-κB targets which help fight infection [27]. In inflammatory responses, these peptides are considered to aid in activating and recruiting immune cells (reviewed in [28]). While both NF-κB pathways are activated upon eye-specific expression of *SCA3*^*polyQ78*^, activation of Relish was stronger: AMPs specific for Relish (*CecA* and *DptA*) were upregulated more than Dif/Dorsal-specific AMPs (*IM1* and *IM2*; Fig. 2a and shown in [29]). Activation of Relish in SCA3^polyQ78^-expressing eyes occurred in astrocytes: GFP under control of the promoter of a Relish-dependent gene was expressed predominantly in the astrocytes that were present in SCA3^polyQ78^-expressing eyes (Additional file 4a). In control eyes, no induction of GFP was seen.

We further focused on the cell-non-autonomous role of astrocyte-specific Relish signaling in SCA3. We first confirmed the results of our screen by using two independent *Relish* RNAi constructs (efficacy of knockdown, see Additional file 4b) that both decreased *SCA3*^*polyQ78*^-induced degeneration (Fig. 2b). Inversely, the opposite effect was seen upon overexpression of *Relish*. Modulation of *Relish* expression in astrocytes did not affect morphology of control eyes (Additional file 4c). In flies heterozygous for *Relish*, degeneration was attenuated, similar to astrocyte-specific *Relish* RNAi. The effect of Relish in astrocytes on eye degeneration was quantified (Fig. 2c) by determining the fraction of the eyes containing necrotic spots. Importantly, the effects of Relish on degeneration are cell-non-autonomous, mediated via astrocytes: when coexpressing *SCA3*^*polyQ78*^ and RNAi constructs targeting Relish in the eyes, eye degeneration was not attenuated (Additional file 4d).

When membrane-targeted mCD8-GFP was used as a readout for degeneration [23] in eyes expressing SCA3^polyQ78^, the protective or enhancing effects of the astrocyte-specific down- and upregulation of Relish levels were confirmed (Fig. 2d for GFP images of different genotypes). To see whether modulating of *Relish* expression in astrocytes affected the localization of astrocytes, we coexpressed myr-RFP in astrocytes. We did not see an effect of modulation of *Relish* expression on the localization of the RFP signal (Fig. 2d for RFP images of different genotypes, Fig. 2f for western blot analysis of lysates). These data suggest that Relish in astrocytes does not have an effect on the localization of astrocytes in SCA3^polyQ78^-expressing eyes (additional images are provided in Additional file 4e). We quantified the effect of modulating *Relish* expression in astrocytes on mCD8-GFP levels in SCA3^polyQ78^-expressing eyes by western blot (Fig. 2e). GFP levels in flies heterozygous for Relish were similar to those of Relish RNAi in astrocytes, suggesting the importance of astrocyte-specific Relish signaling in *SCA3*^*polyQ78*^-induced degeneration. The effect of Relish on mCD8-GFP levels in the eye is specifically related to *SCA3*^*polyQ78*^-induced degeneration, as in control flies (not expressing *SCA3*^*polyQ78*^) GFP levels were similar, irrespective of Relish expression (Additional file 4f). Together, these experiments demonstrate that in our SCA3 model, Relish is activated (and Dif/Dorsal to a lesser extent) and that Relish signaling in astrocytes enhances SCA3^polyQ78^-induced degeneration. Thus, eye-specific expression of *SCA3*^*polyQ78*^ promoted Relish activation in astrocytes, and this Relish activation enhanced degeneration in a cell-non-autonomous manner.

**Fig. 3:**
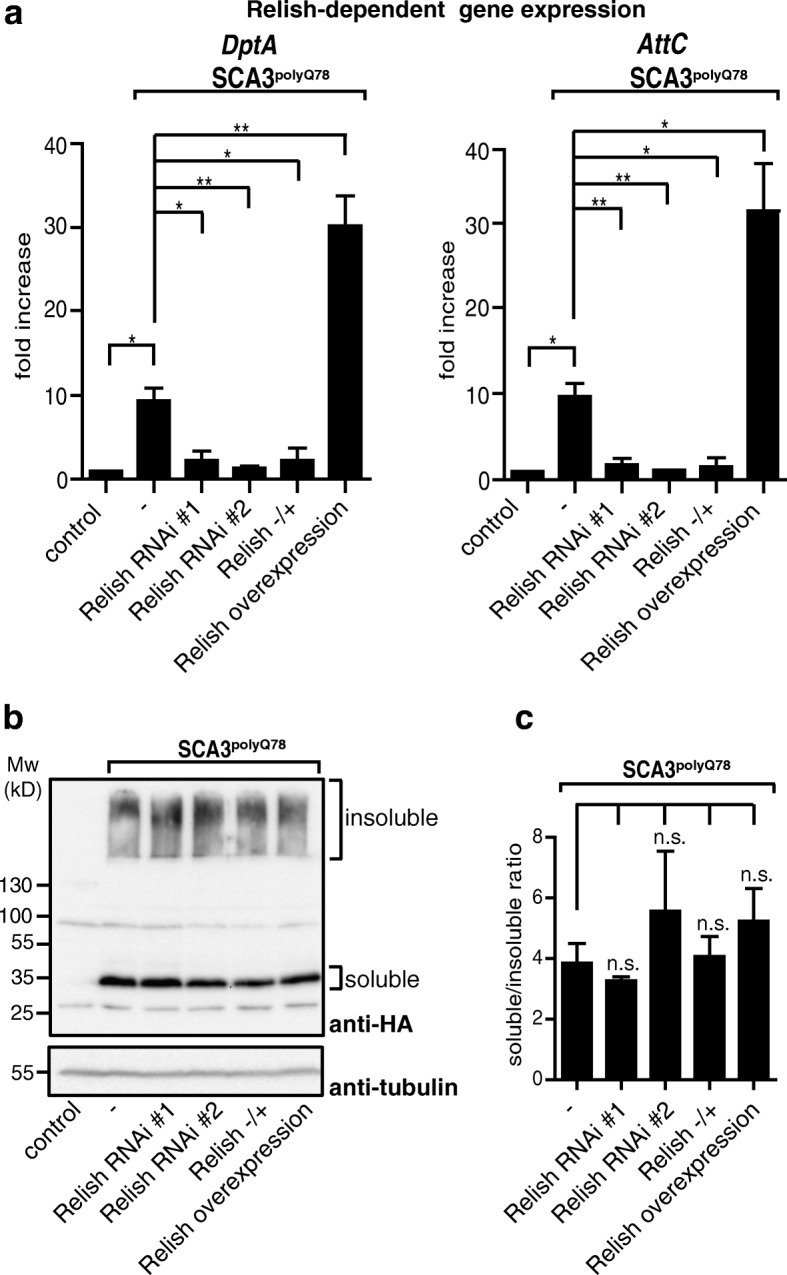
Relish signaling in astrocytes influences SCA3^polyQ78^-induced gene expression but not the extent of SCA3 aggregation. **a** Analysis of the effect of modulating *Relish* expression in astrocytes or Relish heterozygosity on Relish activity in heads of flies expressing *SCA3*^*polyQ78*^ in the eyes. Expression of Relish target genes (*DptA* or *AttC*) was determined in heads of control flies (control) and flies expressing SCA3^polyQ78^ in the eyes. The effect of *Relish* RNAi targeted to astrocytes (*Relish* RNAi #1 and *Relish* RNAi #2), *Relish* overexpression or heterozygosity for *Relish* (*Relish −/+*) in flies expressing eye-specific SCA3^polyQ78^ was determined. **b** Lysates of fly heads described in (**a**) were analyzed on Western blot to determine levels of soluble and aggregated HA-tagged SCA3^polyQ78^. **c** Quantification of the SDS-soluble/SDS-insoluble ratio of SCA3^polyQ78^ of three independent experiments shown in **b**. n.s., not significant. Genotypes in **a** and **b**: control, GMR-QF/+; for other genotypes, see Fig. 2b

In the brain, Relish is activated in glia in aging flies as well as in flies that are mutant for *A-T mutated* (*ATM*), a kinase associated with ensuring genomic integrity and neurodegeneration [30, 31]. We analyzed contribution of astrocytes to overall Relish signaling in the fly head induced by eye-specific expression of *SCA3*^*polyQ78*^. For this, we examined levels of Relish-dependent AMPs in fly heads expressing *SCA3*^*polyQ78*^ in the eyes together with astrocytes-specific targeting of *Relish* RNAi or *Relish* overexpression constructs, or in *SCA3*^*polyQ78*^-expressing flies heterozygous for Relish. Expression of *Relish* RNAi constructs in astrocytes in flies expressing eye-specific *SCA3*^*polyQ78*^ resulted in decreased expression of Relish-specific AMPs (*DptA* and *AttC*) in the head to levels comparable to flies not expressing *SCA3*^*polyQ78*^ (Fig. 3a), whereas overexpression of *Relish* enhanced expression. SCA3 flies heterozygous for *Relish* expressed levels of Relish target genes comparable to those expressing *Relish* RNAi constructs in the astrocytes. Dif/Dorsal-specific target genes were unaffected by \modulating Relish levels in astrocytes (Additional file 5). These data highlight the importance of Relish signaling in astrocytes regarding Relish-dependent immune signaling in the head.

The presence of aggregated or misfolded proteins is toxic to neurons [32]. Possibly, astrocytes could have an effect on levels of protein aggregates. To see whether Relish-specific signaling in astrocytes could have an effect on solubility of SCA3^polyQ78^ in the eyes, we collected heads of flies expressing eye-specific *SCA3*^*polyQ78*^ 2 days after eclosion and analyzed the cell extract. Modulating *Relish* expression in astrocytes or heterozygosity for *Relish* did not affect total levels or solubility of SCA3^polyQ78^ (Fig. 3b, quantification of three independent experiments in Fig. 3c). This suggests that the effect of Relish on SCA3^polyQ78^-induced degeneration is not the result of either a clearance of SCA3^polyQ78^ aggregates or of a shift of the balance of soluble-insoluble SCA3^polyQ78^ towards more soluble SCA3^polyQ78^.

**Fig. 4:**
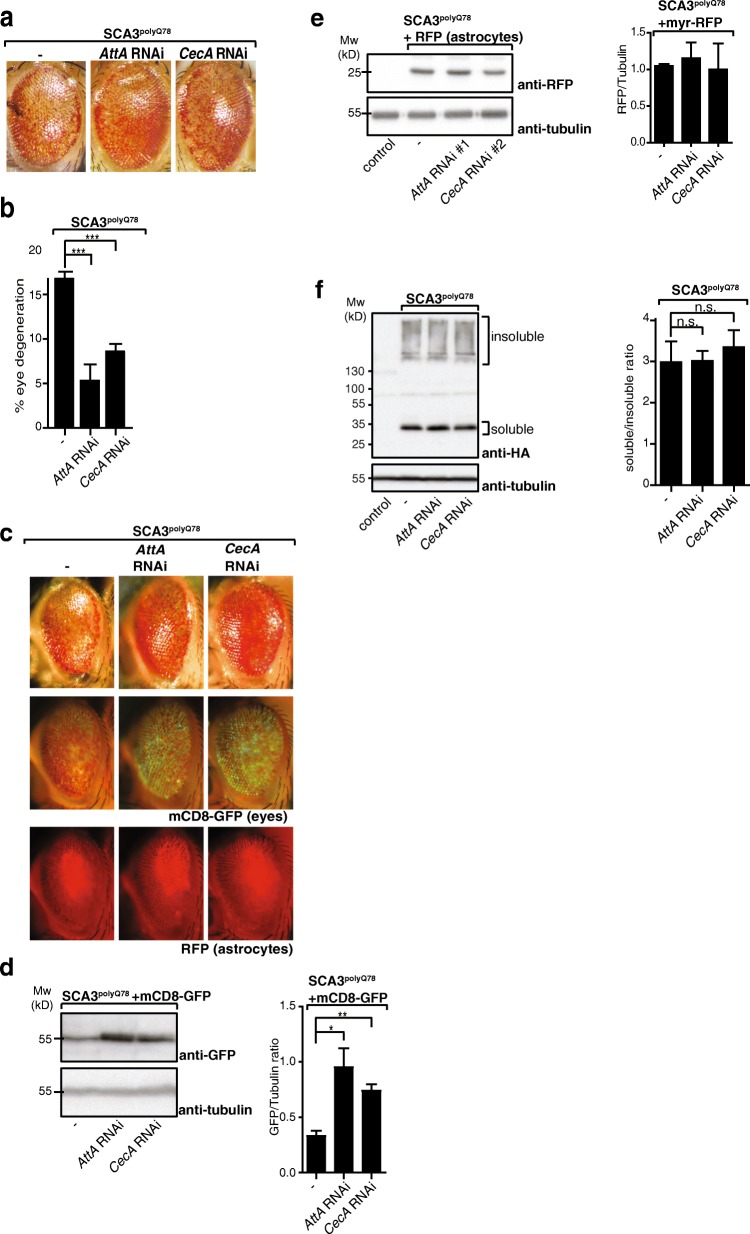
Relish target genes influence SCA3^polyQ78^-induced degeneration but not SCA3^polyQ78^ aggregation or SCA3^polyQ78^-induced relocation of astrocytes. **a** Representative images of flies expressing eye-specific SCA3^polyQ78^ compared to flies coexpressing RNAi constructs in astrocytes targeting Relish target genes *AttA* or *CecA*. **b** Quantification of the fraction of degeneration of the eyes shown in **a**. Data are representative of at least three independent experiments. **c** Representative images of the effect of downregulation of Relish target genes *AttA* or *CecA* in astrocytes on SCA3-induced degeneration or the relocation of astrocytes upon expression of eye-specific SCA3^polyQ78^ by using eye-specific expression of mCD8-GFP and astrocyte-targeted expression of myr-RFP (RFP). **d** Quantification of the effect of Relish target genes in astrocytes on SCA3-induced degeneration by using membrane-targeted mCD8-GFP. Lysates of fly heads expressing mCD8-GFP and SCA3^polyQ78^ in the eyes were analyzed on western blot and compared to flies coexpressing RNAi constructs targeting *AttA* or *CecA* in astrocytes. Quantifications are of at least three independent experiments. **e** No effect on Relish target genes on levels of astrocytes expressing myr-RFP. Lysates of fly heads of control flies or flies expressing myr-RFP in astrocytes together with *SCA3*^*polyQ78*^ in the eyes in the presence or absence or *AMP* RNAi constructs (*AttA* RNAi or *CecA* RNAi) were analyzed on western blot for expression for myr-RFP and tubulin. Quantifications are of two independent experiments. **f** Analysis of the effect of Relish target genes in astrocytes on SDS-solubility of SCA3^polyQ78^ expressed in the eyes. Head lysates of flies expressing HA-tagged SCA3^polyQ78^ in the eyes were compared to flies coexpressing RNAi constructs targeting *AttA* or *CecA* in astrocytes. Quantifications of the SDS-soluble/SDS-insoluble ratio are of at least three independent experiments. Genotypes **a** -: *GMR*-QF2/+; *QUAS-SCA3*^*polyQ78*^*:: alrm-Gal4/+. AttA* RNAi, *GMR-QF2/+*; *QUAS-SCA3*^*polyQ78*^*:: alrm-Gal4/UAS-AttA RNAi*. *CecA* RNAi, *GMR-QF2/+*; *QUAS-SCA3*^*polyQ78*^*:: alrm-Gal4/+*; *UAS-CecA RNAi/+*. **c**, **d** Con: *GMR-QF2/+*, the rest of the genotypes as in **a** but with coexpression of *QUAS-mCD8-GFP* or *UAS-myr-RFP.* **e, f** Con: *GMR-QF2/+*; other samples as in **a**

To see whether Relish can modulate SCA3-induced eye degeneration via Relish-dependent AMPs, we expressed RNAi constructs targeting two Relish-dependent AMPs (*AttA* and *CecA*; efficacy of knockdown in Additional file 6a). Astrocyte-specific downregulation of these AMPs attenuated the degenerative SCA3 eye phenotype (Fig. 4a; quantification of degeneration in Fig. 4b), similar to decreasing Relish activity in astrocytes (Fig. 2a, b). No effect on eye morphology was seen when RNAi targeting AMPs was expressed in astrocytes (Additional file 6b).

We next analyzed mCD8-GFP levels in the eyes as a measure of degeneration in SCA3^polyQ78^-expressing eyes and examined the effect of astrocyte-specific knockdown of Relish-specific AMPs. Levels of mCD8-GFP were increased upon knockdown of AMPs, demonstrating attenuation of SCA3^polyQ78^-induced eye degeneration (Fig. 4c; analysis on western blot and quantification of three independent experiments in Fig. 4d). In control eyes, no effects of downregulation of Relish-specific AMPs expression in astrocytes on mCD8-GFP levels in the eyes were found (Additional file 6c). We also examined whether astrocyte-specific knockdown of Relish-specific AMPs could influence the presence of astrocytes in SCA3^polyQ78^-expressing eyes, but did not see an effect (Fig. 4c), and the numbers of astrocytes were also not affected, as analyzed by levels of myr-RFP in astrocytes (Fig. 4e).

When we examined the effect of astrocyte-specific downregulation of AMPs on SCA3^polyQ78^ levels or solubility, we did not observe an effect (Fig. 4f; quantification of three independent experiments), similar to modulating Relish levels in astrocytes. These data show that the effect of Relish on degeneration in our SCA3 model is, to a significant extent, mediated via the generation of AMPs, and that the effect of AMPs does not occur via alterations in SCA3^polyQ78^ solubility.

**Fig. 5:**
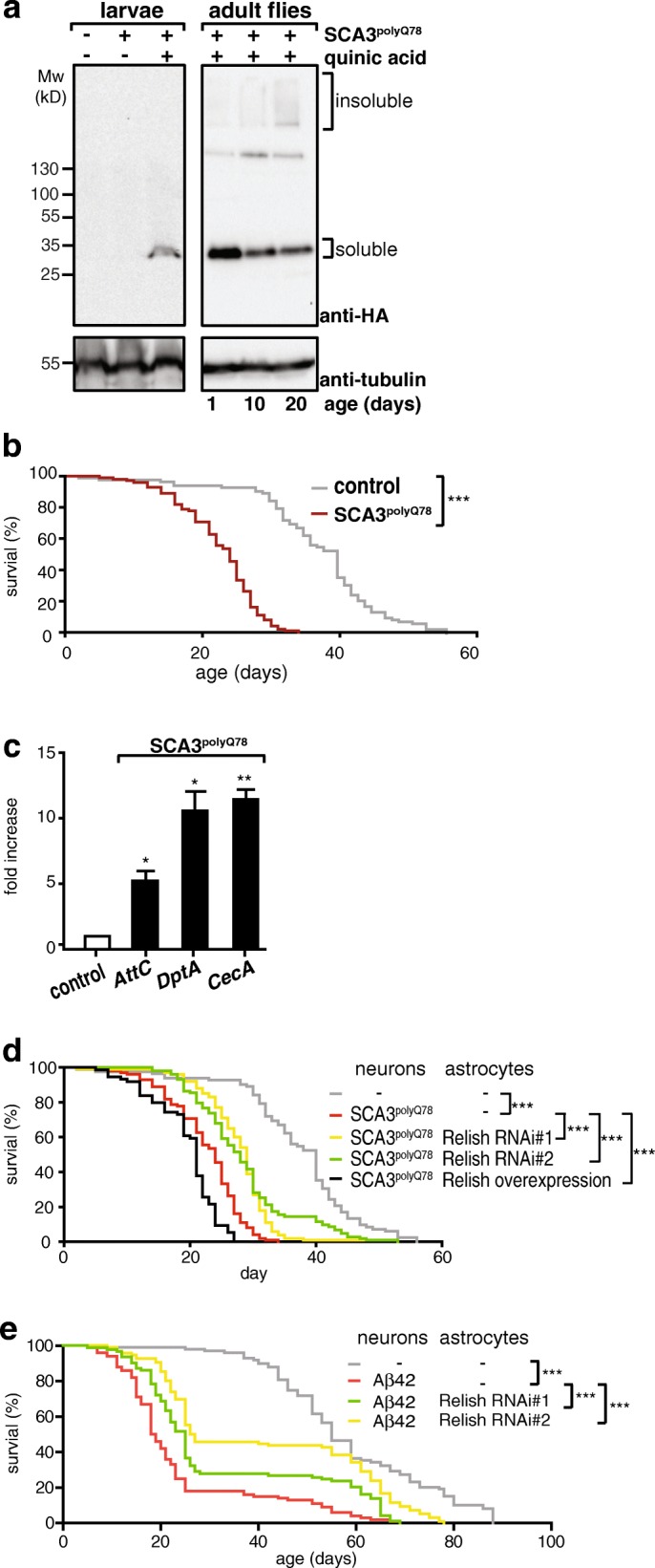
Analysis of SCA3^polyQ78^ expression in neurons and the effect of Relish in astrocytes on neuronal expression of SCA3^polyQ78^ or Abeta42. **a** Inducible expression of *SCA3*^*polyQ78*^ in neurons can be suppressed during development. Control larvae or larvae expressing inducible *SCA3*^*polyQ78*^ were cultured on food with and without quinic acid. Flies expressing inducible *SCA3*^*polyQ78*^ were cultured on food with quinic acid for the times indicated to induce *SCA3*^*polyQ78*^ expression. Levels and aggregation of HA-tagged *SCA3*^*polyQ78*^ were determined in lysates of larvae or fly heads on western blot. Tubulin was used to verify equal loading. **b** Effect of inducible *SCA3*^*polyQ78*^ on lifespan. Control flies or 4-day-old flies expressing inducible *SCA3*^*polyQ78*^ were cultured on food containing quinic acid and the lifespan was analyzed. **c** Expression of Relish target genes *AttC, DptA* and *CecA* was analyzed in heads of 15-day-old flies expressing *SCA3*^*polyQ78*^ in neurons. **d** Analysis of modulation of *Relish* expression specifically in astrocytes on the lifespan of flies expressing *SCA3*^*polyQ78*^ in neurons. Flies expressing *SCA3*^*polyQ78*^ in neurons induced as in **b** were compared to flies coexpressing astrocyte-targeted *Relish* RNAi (Relish RNAi #1 or #2) or overexpressing *Relish* in astrocytes. To induce changes in *Relish* expression only in adult flies, crosses were kept at 18 °C. 4-day-old progeny were shifted to 25 °C to allow expression of *Relish* constructs in astrocytes. **e** Downregulation of *Relish* expression in astrocytes extends lifespan in an Alzheimer model. Lifespan of control flies and flies expressing Abeta42 in neurons were compared to flies expressing Abeta42 (Aβ42) in neurons and *Relish* RNAi constructs in astrocytes. Induction of *Relish* RNAi was done as in **d**. Genotypes: **a** -, *tub-QS/+*; *alrm-Gal4/+*; *nSyb-QF2::tub-QS/+*. *SCA3*^*polyQ78*^, *tub-QS/+*; *alrm-Gal4::QUAS-SCA3*^*polyQ78*^*/+*; *nSyb-QF2::tub-QS/+*. **b**, **c** Control, *tub-QS/+*; *alrm-Gal4/+*; *nSyb-QF2::tub-QS/+*. *SCA3*^*polyQ78*^, *tub-QS/+*; *alrm-Gal4::QUAS-SCA3*^*polyQ78*^*/+*; *nSyb-QF2::tub-QS/+*. **d** -, *tub-QS/+*; *alrm-Gal4/+*; *nSyb-QF2::tub-QS/+. SCA3*^*polyQ78*^, *tub-QS/+*; *alrm-Gal4::QUAS-SCA3*^*polyQ78*^*/+*; *nSyb-*QF*::tub-QS/+*; Relish RNAi #1, Relish RNAi #2, or Relish overexpression, as in SCA3^polyQ78^, but with expression of *UAS-Relish RNAi #1* or #2 or *UAS-GFP-Relish* in astrocytes. **e** -, *alrm-Gal4/+*; *nSyb-QF2*. Aβ42: *alrm-Gal4::QUAS-Abeta42/+*; *nSyb-QF2*/+. Relish RNAi (#1 or #2), *alrm-Gal4:: QUAS-Abeta42/UAS-Relish RNAi #1 or #2)*; *nSyb-QF2/+*

We next tested whether the observed effects of the Relish pathway in astrocytes on (short term) degeneration also translates into a (long term) survival benefit in flies expressing *SCA3*^*polyQ78*^ in neurons. Astrocyte-specific Relish inhibition may also extend lifespan in neurons expressing *SCA3*^*polyQ78*^, given our observations of Relish in astrocytes on the SCA3 eye phenotype. We expressed *SCA3*^*polyQ78*^ pan-neuronally (not in astrocytes) and examined effects of modulation of Relish expression specifically in astrocytes (specificity of expression shown in Additional file 7a) using the following two independent binary expression systems (Additional file 7b): UAS-Gal4 and QUAS-QF2. To avoid potential detrimental effects of *SCA3*^*polyQ78*^ during development, we expressed *SCA3*^*polyQ78*^ in adult flies only, using the inducible “Q system,” in which the neuronally expressed transcription factor (QF2) that induces *SCA3*^*polyQ78*^ expression is coexpressed with QF-suppressor QS [20] (Additional file 7b). This way, expression of *SCA3*^*polyQ78*^ is suppressed during development (Fig. 5a). Feeding quinic acid suppresses QS, thus alleviating inhibition of QF2 resulting in the neuron-specific expression of SCA3^polyQ78^, of which the extent of aggregation increased over time (Fig. 5a). The lifespan of flies expressing SCA3^polyQ78^ in neurons was significantly shortened (Fig. 5b). Quinic acid by itself did not affect lifespan (Additional file 7c), excluding non-specific effects. Relish-dependent gene expression was increased in flies that expressed SCA3^polyQ78^ in neurons (Fig. 5c), similar to expression of SCA3^polyQ78^ in the eyes (Fig. 2a).

We next wished to examine whether modulating Relish levels in astrocytes in adult flies could have an effect on lifespan of flies that expressed *SCA3*^*polyQ78*^ pan-neuronally. For this, levels of Relish were modulated in astrocytes of adult flies to exclude effects on development. Hereto, we used the transcription factor Gal4 to drive the expression of the Relish constructs. At a lower temperature (18 °C), activity of Gal4 is low and *Relish* RNAi constructs do not decrease *Relish* expression (Additional file 7d). 4 days after eclosion, we induced *SCA3*^*polyQ78*^ expression in neurons by adding quinic acid to the food. Flies were shifted to 25 °C to induce expression of the Relish constructs in astrocytes. Importantly, reducing Relish expression in astrocytes extended the lifespan of SCA3 flies, whereas overexpression shortened the lifespan (Fig. 5d). In control flies, downregulating *Relish* expression in astrocytes had no effect on lifespan (Additional file 7e), indicating that the effects of *Relish* RNAi are linked to SCA3^polyQ78^-mediated degeneration of neurons. However, overexpression of *Relish* alone in astrocytes significantly shortened lifespan (Additional file 7e), precluding analysis on Relish overexpression on SCA3^polyQ78^-mediated shortening of lifespan.

In parallel, SCA3-related effects on motor function were analyzed, measured as climbing ability. The SCA3^polyQ78^-induced decrease in climbing ability was partially alleviated by knockdown of *Relish* in astrocytes (Additional file 7f). As was observed with modulating *Relish* expression in astrocytes in SCA3^polyQ78^-expressing eyes, no effects of Relish in astrocytes on total brain SCA3^polyQ78^ aggregation load or levels could be detected (Additional file 7g).

The effects of Relish modulation on SCA3-related degeneration were not related to alterations of the levels of *SCA3*^*polyQ78*^ aggregates (Fig. 3b and Additional file 7g). Therefore, we hypothesized that modulation of the Relish pathway in astrocytes could be more generic and relevant to a neurodegenerative disease associated with the presence of extraneuronal aggregates, such as in Alzheimer’s disease. For this, we turned to an established *Drosophila* model of Alzheimer’s disease [18], in which the Amyloid beta (Abeta42; Aβ42) peptides are fused to a secretion signal peptide (of the *necrotic* gene), resulting in the secretion of Aβ42 peptides. When expressed in neurons (Additional file 7h), these Aβ42 peptides are secreted and form extracellular aggregates in flies, reminiscent of the plaques in human AD [33]. In this AD fly model, flies displayed progressive neurodegeneration, with mobility and memory deficits, and reduced lifespan [18, 34]. In line with our hypothesis, the lifespan of flies expressing Aβ42 in neurons was shortened, but extended when *Relish* expression was suppressed in astrocytes, using two independent RNAi lines targeting *Relish* (Fig. 5e). This, together with the data on SCA3, demonstrates that modulating Relish signaling in astrocytes has beneficial effects in neurodegeneration, irrespective of the aggregation process that initiates it.

Our data show that astrocytes can modulate the rate of neurodegenerative disease progression in a cell-non-autonomous manner. This is the first time to show that neuron-specific expression of a pathological, degeneration-inducing protein results in activation of astrocytes, which can subsequently influence symptoms of degeneration.

## Discussion

Astrocytes become reactive during the course of a number of age-related neurodegenerative diseases associated with aggregates [6], and several reports have suggested that during the course of disease astrocytes gain neurotoxic properties ([4] reviewed in [6]). However, potential mechanisms for how astrocytes affect progression of disease have yet been lacking, in part due to the fact that in most studies disease-causing genes are expressed in both neuronal and non-neuronal cells in the brain. Expression of aggregation-prone proteins in astrocytes can cell-non-autonomously influence neuronal viability [5], reviewed in [6]. However, putative cell-non-autonomous contributions of astrocytes to neurodegeneration in response to neurons expressing aggregation-prone proteins are unclear. Here, we investigate how signaling in astrocytes can influence neurons that express aggregation-prone, disease-causing proteins, thus investigating cell-non-autonomous contributions of astrocytes to neurodegeneration. We identified astrocytes in *Drosophila* eyes expressing SCA3^polyQ78^ but not in control eyes or eyes expressing SCA3^polyQ27^ (Fig. 1d), suggesting a role for astrocytes. We further investigated putative cell-non-autonomous contributions of genes in astrocytes in a candidate RNAi screen, where the effect of RNAi-mediated downregulation of individual genes in astrocytes on SCA3^polyQ78^-induced eye degeneration was determined. This resulted in the identification of astrocyte-specific enhancers and suppressors of the SCA3-related neurodegeneration (Additional file 3). These results demonstrate, for the first time, the importance of signaling in astrocytes induced by specific expression of aggregation-prone, neurodegeneration-associated proteins. Astrocytes in *Drosophila* share structural and functional similarities with mammalian astrocytes: amongst others, they provide trophic support to neurons and are involved in neurotransmitter recycling (reviewed in [35]). Therefore, genes that we identified in our screen may be relevant for mammalian astrocytes as well.

Conserved NF-κB transcription factor Relish was identified as an enhancer in our SCA3 model, and its activity was upregulated in the head upon eye-specific SCA3^polyQ78^ expression. We therefore focused on Relish signaling for more in-depth analyses. Relish was specifically activated in astrocytes present in eyes expressing SCA3^polyQ78^, but not in astrocytes elsewhere in the head (Additional file 4a). Downregulation of expression in astrocytes of both *Relish*, but also Relish-dependent AMPs alleviated SCA3^polyQ78^-induced eye degeneration (Figs. 2 and 4). In contrast, we did not see an effect of downregulation of *Relish* expression on degeneration in eyes coexpressing SCA3^polyQ78^ and *Relish* RNAi constructs (Additional file 4d). Thus, eye-specific expression of aggregation-prone SCA3^polyQ78^ results in cell-non-autonomous activation of NF-κB in astrocytes. We next investigated possible cell-non-autonomous contributions of astrocytes to neurodegeneration.

Downregulation of *Relish* expression in astrocytes not only delayed neurodegeneration (suggested by enhanced mobility, Additional file 7f) and extended lifespan of flies expressing *SCA3*^*polyQ78*^ in neurons (Fig. 5d), but also extended lifespan in flies expressing neuronal Aβ42 peptides (Fig. 5e). We did not see an effect of downregulating *Relish* expression in astrocytes on lifespan (Additional file 7e), underscoring the efficacy of using this pathway as a target to inhibit the detrimental effects of SCA3^polyQ78^. However, overexpression of Relish in astrocytes was sufficient to shorten lifespan, demonstrating toxic effects of Relish. This is in agreement with earlier studies demonstrating that expression of Relish or Relish-specific AMPs was sufficient to induce neurodegeneration [30, 36]. Of particular interest is that in aging flies Relish activity and levels of AMPs were increased in both neurons and glia, and constitutes an important determinant of lifespan control and the onset of neurodegeneration [31]. In addition to this, our data demonstrate the importance of cell-non-autonomous Relish signaling in astrocytes in neurodegeneration associated with aggregates, which is generally aging-associated. The presence of aggregation-prone, neurodegeneration-associated proteins in neurons resulted in Relish signaling in astrocytes, which subsequently influenced neuronal functioning. Together, these findings suggest the importance of NF-κB activity in astrocytes in age-associated neurodegenerative diseases.

Our data thus provide direct evidence for earlier suggestions that astrocytes are not only activated in response to neurodegeneration (as, e.g., demonstrated by elevation of GFAP, glial fibrillary acidic protein, a marker for astrocyte activation) [5], but indeed can modulate disease progression. The relevance of the cell-non-autonomous Relish activation in astrocytes is underscored by the observation that downregulation of Relish in astrocytes can influence disease progression. A toxic gain of function in astrocytes was also demonstrated in a mice expressing human aggregation-prone TAR DNA-binding protein 43 (TDP-43) in neurons [37], resulting in alterations in protein expression in astrocytes. Depletion of an upregulated, astrocyte-specific factor reduced neurotoxicity, underscoring the importance of astrocytes to neurotoxicity. Our findings that genes in the NF-κB pathway are key mediators of such a response extends the observation that astrocyte-specific, transient expression of IKK (IκB kinase 2), resulted in neurodegeneration in mice [38].

While Lattke et al. showed sufficiency of astrocyte-specific NF-κB signaling in inducing neurodegeneration, our data now demonstrate that signals from neurons expressing aggregation-prone proteins suffice to induce cell-non-autonomous NF-κB activation in astrocytes. The importance of NF-κB activation in astrocytes was suggested by date presented in Fig. 3a and Additional file 5a. The presence of astrocytes in *SCA3*^*polyQ78*^-expressing eyes (Fig. 1d, Additional file 4) suggests recruitment of astrocytes, as shown for Alzheimer’s disease (reviewed in [39]). Downregulation of NF-κB in astrocytes did not appear to alter the presence of astrocytes in SCA3^polyQ78^-expressing eyes (Fig. 2d, Additional file 4e), suggesting that additional signaling in astrocytes promotes the recruitment of astrocytes to degenerating cells. Together, these data demonstrate the existence and importance of intercellular signaling between neurons and astrocytes as an important determinant for the speed of neurodegeneration.

The nature of the neuronal signals that trigger NF-κB activation in astrocytes remains to be elucidated. Possibly, damage-associated molecular patterns (DAMPs), including *SCA3*^*polyQ78*^ aggregates, released from neurons can result in activation of NF-κB in astrocytes. It is unlikely that the Relish canonical pathway, headed by PGRP-LC (reviewed in [27]), accounts for Relish activation, since modulation of PGRP-LC did not significantly affect the SCA3 eye phenotype (Additional file 1). Previous reports have demonstrated Relish activation independent of PGRP-LC, either by neuropeptides or nitric oxide [40, 41], which may account for Relish activation in our model.

Whereas we show that astrocytes can directly modulate neuronal health, this appeared not to be associated with alteration in the burden of protein aggregates in the brain. However, decreasing the levels of misfolded or aggregated proteins has been suggested as a therapeutic strategy [42]. Furthermore, reduction of oxidative damage can decrease degeneration [43]. Thus, a combination of therapeutic strategies targeting aggregates, astrocyte-mediated neurotoxicity as well as oxidative damage may have optimal effects in alleviating aggregates-associated neurodegeneration.

Targeting NF-κB signaling in astrocytes to alleviate neurodegeneration may be more broadly applicable: the lifespan of flies expressing Aβ peptides, present in patients with AD, was extended upon astrocyte-specific inhibition of NF-κB (Fig. 5e). Thus, despite intracellular localization of aggregates in SCA3 and extracellular localization of Aβ peptides in AD, astrocyte-specific NF-κB inhibition extended lifespan in fly models for both diseases. This suggests that targeting NF-κB in astrocytes may be beneficial in other aggregates-associated neurodegenerative diseases.

## Conclusion

In conclusion, our data demonstrate that astrocytes respond to cellular stress or damage in neurons induced by neuronal expression of disease-associated, aggregation-prone proteins. These astrocytic responses are important modulators of neurodegeneration. We demonstrate that activation of transcription factor NF-κB Relish occurs in astrocytes in response to proteotoxic stress in neurons, and that in this situation astrocyte-specific inhibition of Relish enhances vitality and extends lifespan. While proteotoxic stress in astrocytes can contribute to neurodegeneration, these data highlight the importance of astrocytic responses to cellular stress or damage in neurons, and provide putative therapeutic potential.

## Additional files

Additional file 1: Genetic setup to analyze cell-non-autonomous or cell-autonomous roles of genes to SCA3. **a** To model SCA3 in *Drosophila* eyes, we used the Q (QUAS-QF) system, in which a *QUAS-SCA3*^*polyQ78*^ (or *QUAS-SCA3*^*polyQ27*^*)* construct was induced by QF2 specifically expressed in the eyes, *GMR-QF2*. To downregulate gene expression in astrocytes, we used the UAS-Gal4 system. *UAS-RNAi* constructs were specifically expressed in astrocytes by using a Gal4 expressed in astrocytes, *alrm*-Gal4. Cell-autonomous roles of genes in SCA3 can be analyzed by coexpressing *UAS-SCA3*^*polyQ78*^ with UAS constructs in the eye, using eye-specific *GMR-Gal4*. **b** Independent expression of the QUAS-QF2 system (GFP in the eyes) and the UAS-Gal4 system (RFP in astrocytes) in late pupa. Genotype, *GMR-QF2/+*; *alrm-Gal4/UAS-myr-RFP*; *QUAS-mCD8-GFP/+.* (PDF 186 kb)

Additional file 2: Analysis of localization of astrocytes induced by eye-specific expression of *SCA3*^*polyQ78*^*.* The eyes of control flies that express myr-RFP in astrocytes in the absence or presence of SCA3^polyQ27^ or SCA3^polyQ78^ were analyzed for expression and localization of RFP. Genotypes: control, *GMR-QF2/+*; *alrm-Gal4::UAS-myr-RFP/+.* SCA3^polyQ27^, *GMR-QF2/+*; *alrm-Gal4::UAS-myr-RFP/QUAS-SCA3*^*polyQ27*^*.* SCA3^polyQ78^, *GMR-QF2/+*; *alrm-Gal4::UAS-myr-RFP/QUAS-SCA3*^*polyQ78*^*.* (PDF 4177 kb)

Additional file 3: RNAi screen to determine the contribution of genes in astrocytes to SCA3^polyQ78^-induced eye degeneration. Flies expressing *SCA3*^*polyQ78*^ in the eyes together with *alrm-Gal4* were crossed to fly lines containing *UAS-RNAi* constructs to specifically knock down genes in astrocytes. Progeny expressing SCA3^polyQ78^ in the eyes with individual genes in astrocytes knocked down were analyzed for SCA3^polyQ78^-induced eye degeneration. The extent of degeneration was quantified and plotted in a table. The percentage of degeneration in SCA3^polyQ78^ eyes was set at 100% and the effect of RNAi-mediated gene knockdown in astrocytes was determined by the percentage of deviation from the control. The suppressors of degeneration (in which RNAi-induced downregulation of gene expression in astrocytes enhanced the SCA3^polyQ78^ phenotype, 60% or more deviation from the control) are shown in orange, the enhancers (40% or more deviation from the control) in purple. Genotype of the crosses SCA3^polyQ78^, *GMR-QF2/+*; *QUAS-SCA3*^*polyQ78*^*:: alrm-Gal4/+.* RNAi: *GMR-QF2/+*; *QUAS-SCA3*^*polyQ78*^*:: alrm-Gal4/+* together with *UAS-RNAi*. (PDF 149 kb)

Additional file 4: **a** Activation of Relish in *SCA3*^*polyQ78*^-expressing eyes predominantly occurred in astrocytes. (top) Representative picture of a control eye expressing GFP under the control of the *Attacin* promoter (*att*-GFP) and astrocytes expressing RFP or an eye coexpressing SCA3^polyQ78^. Pictures were taken from live eyes. (bottom) Representative picture of a dissected eye expressing SCA3^polyQ78^, together with *att*-GFP and RFP-expressing astrocytes. Nuclei are stained with Hoechst (blue). Note that in the bottom panel there is only an overlap between the RFP and GFP signal in the ommatidia. This may cause the imperfect overlap between the RFP and GFP signal in SCA3^polyQ78^-expressing flies in the top panel: the RFP signal from the underlying astrocytes. **b** Efficacy of *Relish* knockdown. Flies expressing *daughterless-Gal4* (*da-Gal4*, ubiquitously expressed) were crossed to control flies (w1118) or fly lines containing RNAi constructs targeting *Relish* (Relish RNAi #1 and Relish RNAi #2) and expression of *Relish* in the progeny was determined. **c** Modulation of expression in astrocytes does not affect morphology of control eyes. Control flies or fly lines containing RNAi constructs targeting *Relish* (Relish RNAi #1 and Relish RNAi #2) or expressing *Relish* were crossed to *alrm-Gal4* flies, and the morphology of the eyes was determined. **d** No cell-autonomous effect of Relish on the degenerative SCA3^polyQ78^ eye phenotype. Constructs targeting *Relish* were coexpressed in the eyes expressing SCA3^polyQ78^. **e** Expression of *Relish* RNAi in astrocytes does not influence the relocation of the astrocytes to the eye induced by eye-specific expression of SCA3^polyQ78^. Images of multiple flies to show similar levels of RFP in the eyes between flies expressing eye-specific of SCA3^polyQ78^ and astrocyte-specific myr-RFP (RFP) in the absence or presence of *Relish* RNAi targeted to astrocytes. **f** Modulating *Relish* expression in astrocytes does not affect levels of mCD8-GFP in the eyes. Lysates of fly heads expressing eye-specific mCD8-GFP were compared to fly heads expressing eye-specific mCD8-GFP together with astrocyte-specific knockdown or overexpression of Relish constructs or flies heterozygous for Relish (Relish −/+). Analysis of the mCD8-GFP/tubulin ratio of two independent experiments is shown below. Genotypes **a** control, *GMR-QF2/+*; *alrm-Gal4::UAS-myr-RFP*; *att-GFP/+.* SCA3^polyQ78^, *GMR-QF2/+*; *alrm-Gal4::UAS-myr-RFP::QUAS-SCA3*^*polyQ78*^; *att-GFP/+.* **b** Control, *da-Gal4/+*. Relish RNAi #1, *UAS-Relish RNAi #1/+*; *da-Gal4/+*. Relish RNAi #2, *UAS-Relish RNAi #2/+*; *da-Gal4/+*. **c** Control, *alrm-Gal4/+*. Relish RNAi #1, *alrm-Gal4/UAS-Relish RNAi#1.* Relish RNAi #2, *alrm-Gal4/+*; *UAS-Relish RNAi#2/+.* GFP-Relish*: alrm-Gal4/UAS-GFP-Relish*. **d** -, *GMR-Gal4::UAS-SCA3*^*polyQ78*^*/+*. *Relish* RNAi#1, *GMR-Gal4:: UAS-SCA3*^*polyQ78*^*/UAS-Relish RNAi#1*. *Relish* RNAi#2, *GMR-Gal4::UAS-SCA3*^*polyQ78*^*/UAS-Relish RNAi #2*. Relish overexpression, *GMR-Gal4::UAS-SCA3*^*polyQ78*^*/UAS-GFP-Relish.* **e** As in Fig. 2d. **f** -, *GMR*-*QF2/+*; *alrm-Gal4/+ QUAS-mCD8-GFP. Relish* RNAi #1, *GMR*-QF2/+; *alrm*-Gal4/*UAS-Relish RNAi #1*; *QUAS-mCD8-GFP/+*. *Relish* RNAi #2, *GMR-QF2/+*; *alrm-Gal4/UAS-Relish RNAi #2*; *QUAS-mCD8-GFP/+*. Relish−/+, *GMR-QF2/+*; *alrm-Gal4*; *Relish E20/QUAS-mCD8-GFP.* Relish overexpression, *GMR-QF2/+*; *QUAS- SCA3*^*polyQ78*^*:: al*rm-Gal4*/UAS-Relish QUAS-mCD8-GFP.* (PDF 1700 kb)

Additional file 5: Effect of modulating *Relish* levels in astrocytes on Dif/Dl-dependent gene expression in the head. Expression of Dif/Dl target genes (*IM1* or *IM2*) was determined in the heads of control flies (control), flies expressing *SCA3*^*polyQ78*^ in the eyes (-), and the effect of *Relish* RNAi targeted to astrocytes (Relish RNAi #1 and Relish RNAi #2), Relish overexpression, or *SCA3*^*polyQ78*^-expressing flies heterozygous for Relish (*Relish −/+*) on Dif/Dl target gene expression was determined by comparing them to flies only expressing *SCA3*^*polyQ78*^. (PDF 125 kb)

Additional file 6: **a** Efficacy of knockdown of *CeCA* or *AttA* gene expression. Flies expressing *daughterless*-Gal4 (*da*-Gal4) were crossed to control flies (w1118) or fly lines containing RNAi constructs targeting *AttA* or *CecA* and expression of *CeCA* or *AttA* in the adult progeny was determined. **b** Downregulating in astrocytes does not affect eye morphology. Control flies or fly lines containing RNAi constructs targeting *CeCA* or *AttA* were crossed to *alrm-Gal4* flies and the morphology of the eyes was determined. **c** No effect of modulating of Relish-dependent AMP expression in astrocytes on mCD8-GFP levels in the eyes. Flies expressing eye-specific *mCD8-GFP* were compared to flies coexpressing RNAi constructs targeting *AttA* or *CecA* in astrocytes. Genotypes (**a**): control, *da-Gal4/+*. *AttA*, *UAS− AttA/+ da-Gal4/+*; *CecA*, *UAS- CecA/+*; *da-Gal4/+*. (**b**) Control, *alrm-Gal 4/+*. *AttA* RNAi, *alrm-Gal 4/UAS-AttA RNAi. CecA* RNAi, *alrm-Gal 4/+*; *UAS-CecA RNAi/+.* (**c**) As in (**b**), but with coexpression of *QUAS-mCD8-GFP*. (c) Control: *GMR*-QF2/+; *alrm-Gal4/+*; *QUAS-mCD8GFP/+. AttA* RNAi, *GMR-QF2/+*; *alrm-Gal4/UAS-AttA RNA*; *QUAS-mCD8GFP/+*. *CecA* RNAi, *GMR-QF2/+*; *alrm-Gal4/+*; *UAS-CecA RNAi/QUAS-mCD8GFP.* (PDF 289 kb)

Additional file 7: **a** The UAS-Gal4 and the QUAS-QF system function independently. *QUAS-QF*-driven GFP expression specifically in neurons (GFP); *UAS-Gal4*-driven expression of *myr-RFP* specifically in astrocytes (RFP). Nuclei are stained with Hoechst (blue). **b** Genetic setup to inducibly express *SCA3*^*polyQ78*^ in neurons and simultaneously modulate gene expression in astrocytes. *QUAS-QF* was used to express *SCA3*^*polyQ78*^; *UAS-Gal4* was used to modulate expression in astrocytes. Neuronally expressed QF2 (expressed under control of the pan-neuronal *nSyb* promoter) is suppressed by QS (expressed under control of the *tubulin* (*tub*) promoter). This suppression is alleviated by quinic acid, resulting in expression of *SCA3*^*polyQ78*^. For details on the fly lines used, see experimental procedures. **c** Quinic acid does not affect lifespan. Control flies were cultured on standard fly food with or without quinic acid, and the fraction of dead flies was determined over time. **d** Raising flies at 18 °C does not induce expression of *Relish* RNAi constructs. Progeny of flies ubiquitously expressing RNAi constructs targeting *Relish* (Relish RNAi #1 and Relish RNAi #2) raised at 18 °C were analyzed for expression of *Relish* as in Additional file 4b. **e** Effect of modulating expression of *Relish* in astrocytes on lifespan. The lifespan of control flies or flies expressing *Relish* RNAi or overexpression constructs specifically in astrocytes was analyzed. **f** Effect of modulating *Relish* expression in astrocytes on impairment of mobility induced by neuronal *SCA3*^*polyQ78*^ expression. Control flies or flies expressing inducible *SCA3*^*polyQ78*^ together with *Relish* RNAi or Relish overexpression constructs targeted to astrocytes were analyzed for mobility over time. Scoring was done as described in the experimental procedures. **g** Effect of modulating *Relish* expression in astrocytes on SCA3^polyQ78^ levels or aggregation. Head lysates of 15-day-old flies as in Fig. 5c were analyzed for expression of HA-tagged SCA3^polyQ78^ on western blot. Tubulin was used as a control for equal loading. **h** Expression levels of Abeta42. Two independent lines, *QUAS-Abeta42 #5* and *QUAS-Abeta42 #7* were analyzed for expression of Abeta42 using 6E10 antibody. Tubulin was used as a control for equal loading. Genotypes: **a** *alrm-Gal4::UAS-myr-RFP*; *nSyb-QF2/QUAS-mCD8-GFP.* **c** *tub-QS/+*; *alrm-Gal4/+*; *nSyb-QF2::tub-QS/+*. **d** Genotypes as in Additional file 4b. (**e**) -, *tub-QS/+*; *alrm-Gal4/+*; *nSyb-QF2: tub-QS/+*. *Relish* RNAi #1, Relish RNAi #2, or Relish overexpression, as in ‘-‘, but with *UAS-Relish RNAi #1* or *#2* or *UAS-GFP-Relish*. **f** Genotypes as in Fig. 5d. **g** As in e. **h** Control, *nSyb-QF2/+*. Abeta42 #5 or #7, *QUAS-Abeta42/+*; *nSyb-QF2/+.* (PDF 609 kb)

## Abbreviations

AD: Alzheimer’s disease
AMPs: Antimicrobial peptides
HD: Huntington’s disease
NF-κB: Nuclear factor kappa B
PBS: Phosphate-buffered saline
SCA3: Spinocerebellar ataxia type 3
SDS-PAGE: Sodium dodecyl sulfate polyacrylamide gel electrophoresis
