# Supplementary figures and images for: Inhibition of NF-κB in astrocytes is sufficient to delay neurodegeneration induced by proteotoxicity in neurons
Source: J Neuroinflammation. 2018 Sep 11;15:261. doi: 10.1186/s12974-018-1278-2 (PMC6134576; doi:10.1186/s12974-018-1278-2)

a

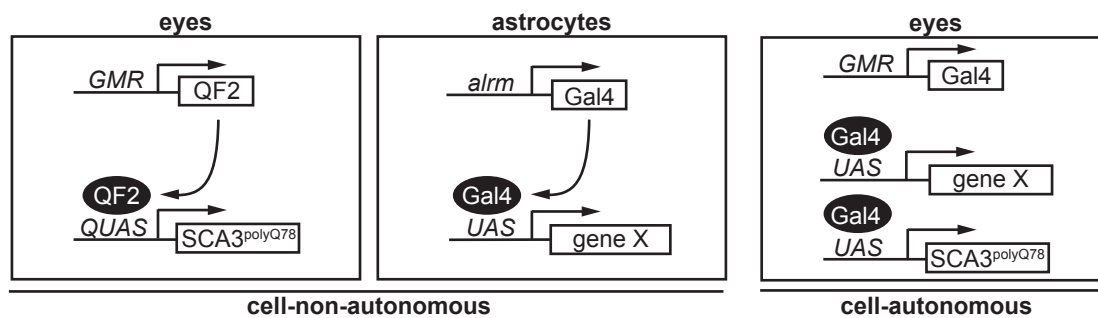

b

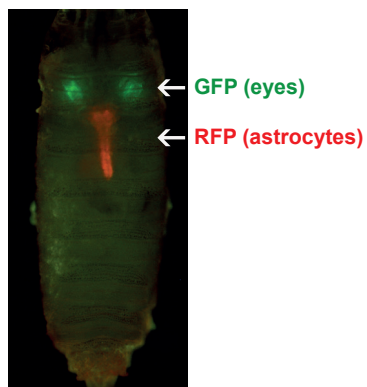

Supplement: Supplementary file 1 — Genetic setup to analyze cell-non-autonomous or cell-autonomous roles of genes to SCA3. a To model SCA3 in Drosophila eyes, we used the Q (QUAS-QF) system, in which a QUAS-SCA3polyQ78 (or QUAS-SCA3polyQ27) construct was induced by QF2 specifically expressed in the eyes, GMR-QF2. To downregulate gene expression in astrocytes, we used the UAS-Gal4 system. UAS-RNAi constructs were specifically expressed in astrocytes by using a Gal4 expressed in astrocytes, alrm-Gal4. Cell-autonomous roles of genes in SCA3 can be analyzed by coexpressing UAS-SCA3polyQ78 with UAS constructs in the eye, using eye-specific GMR-Gal4. b Independent expression of the QUAS-QF2 system (GFP in the eyes) and the UAS-Gal4 system (RFP in astrocytes) in late pupa. Genotype, GMR-QF2/+; alrm-Gal4/UAS-myr-RFP; QUAS-mCD8-GFP/+. (PDF 186 kb) [file 12974_2018_1278_MOESM1_ESM.pdf]

**control**

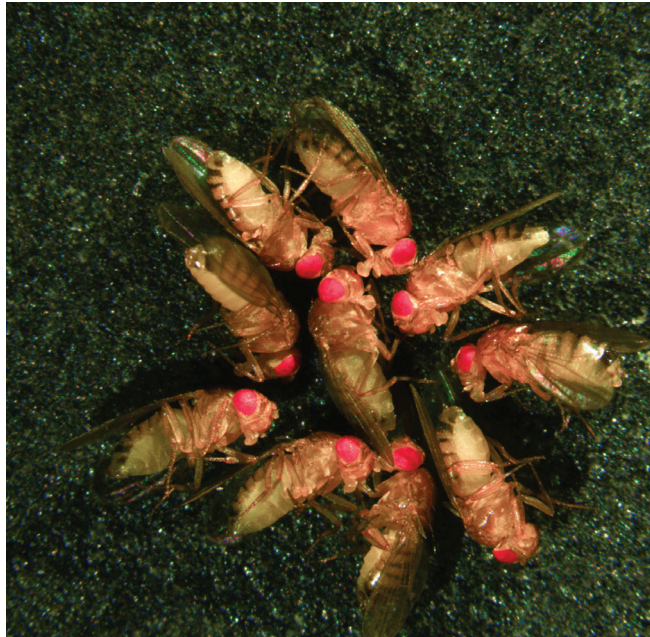

**SCA3<sup>polyQ27</sup>**

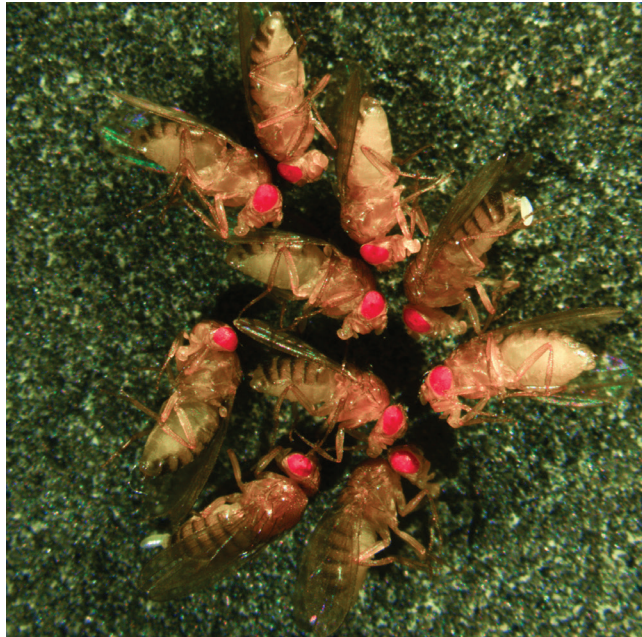

**SCA3<sup>polyQ78</sup>**

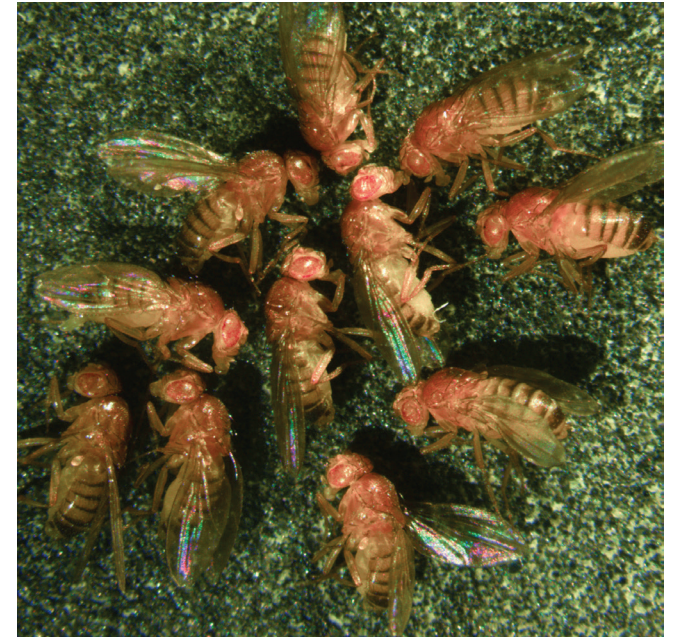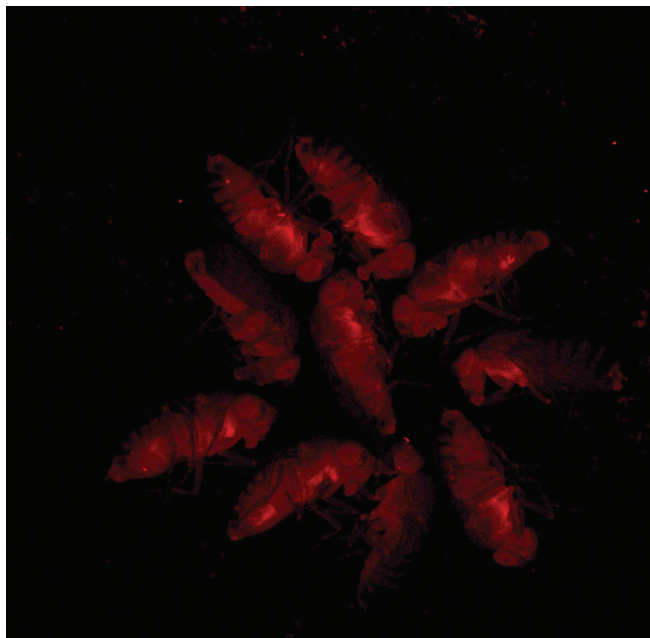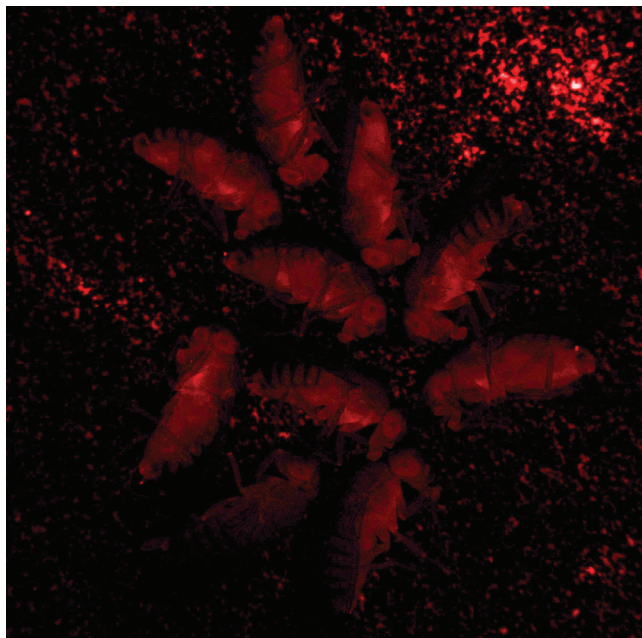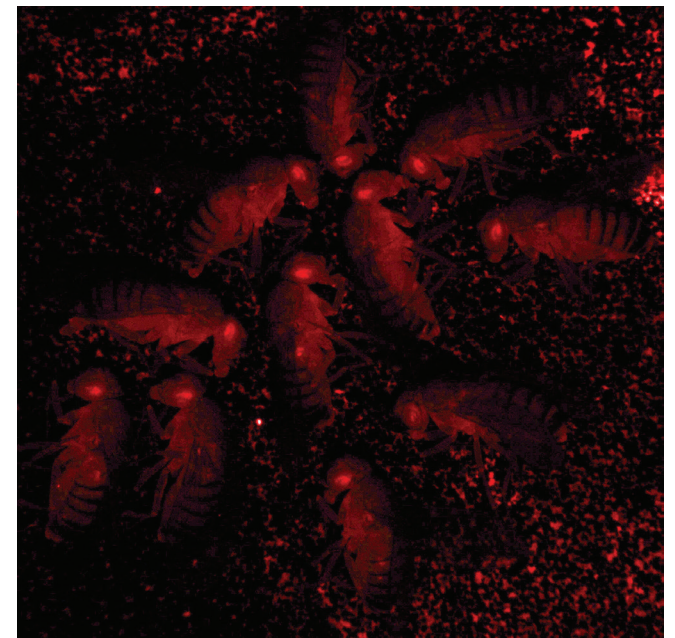

**RFP (astrocytes)**

Supplement: Supplementary file 2 — Analysis of localization of astrocytes induced by eye-specific expression of SCA3polyQ78. The eyes of control flies that express myr-RFP in astrocytes in the absence or presence of SCA3polyQ27 or SCA3polyQ78 were analyzed for expression and localization of RFP. Genotypes: control, GMR-QF2/+; alrm-Gal4::UAS-myr-RFP/+. SCA3polyQ27, GMR-QF2/+; alrm-Gal4::UAS-myr-RFP/QUAS-SCA3polyQ27. SCA3polyQ78, GMR-QF2/+; alrm-Gal4::UAS-myr-RFP/QUAS-SCA3polyQ78. (PDF 4177 kb) [file 12974_2018_1278_MOESM2_ESM.pdf]

# Dif/ Dorsal-dependent gene expression

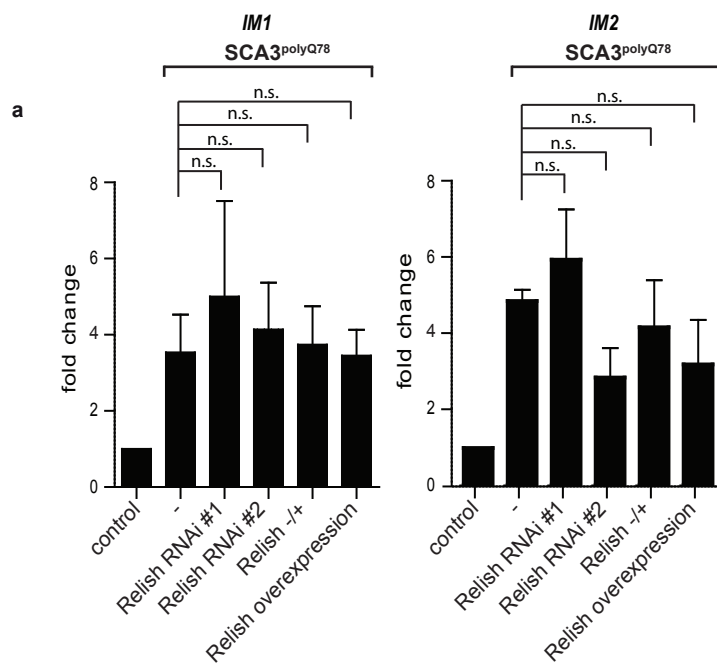

Supplement: Supplementary file 5 — Effect of modulating Relish levels in astrocytes on Dif/Dl-dependent gene expression in the head. Expression of Dif/Dl target genes (IM1 or IM2) was determined in the heads of control flies (control), flies expressing SCA3polyQ78 in the eyes (-), and the effect of Relish RNAi targeted to astrocytes (Relish RNAi #1 and Relish RNAi #2), Relish overexpression, or SCA3polyQ78-expressing flies heterozygous for Relish (Relish −/+) on Dif/Dl target gene expression was determined by comparing them to flies only expressing SCA3polyQ78. (PDF 125 kb) [file 12974_2018_1278_MOESM5_ESM.pdf]

**a**

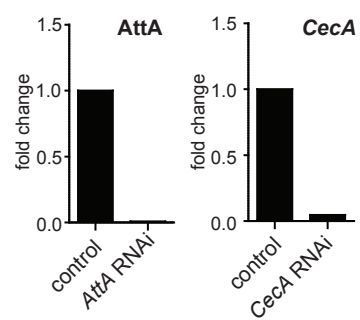

**b**

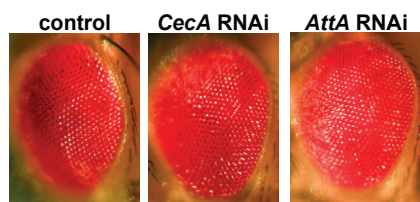

**c**

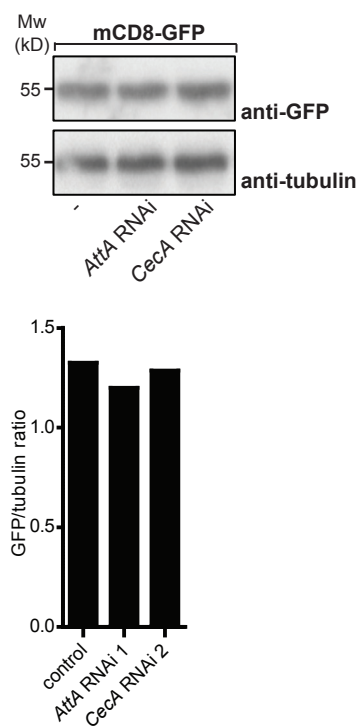

Supplement: Supplementary file 6 — a Efficacy of knockdown of CeCA or AttA gene expression. Flies expressing daughterless-Gal4 (da-Gal4) were crossed to control flies (w1118) or fly lines containing RNAi constructs targeting AttA or CecA and expression of CeCA or AttA in the adult progeny was determined. b Downregulating in astrocytes does not affect eye morphology. Control flies or fly lines containing RNAi constructs targeting CeCA or AttA were crossed to alrm-Gal4 flies and the morphology of the eyes was determined. c No effect of modulating of Relish-dependent AMP expression in astrocytes on mCD8-GFP levels in the eyes. Flies expressing eye-specific mCD8-GFP were compared to flies coexpressing RNAi constructs targeting AttA or CecA in astrocytes. Genotypes (a): control, da-Gal4/+. AttA, UAS− AttA/+ da-Gal4/+; CecA, UAS- CecA/+; da-Gal4/+. (b) Control, alrm-Gal 4/+. AttA RNAi, alrm-Gal 4/UAS-AttA RNAi. CecA RNAi, alrm-Gal 4/+; UAS-CecA RNAi/+. (c) As in (b), but with coexpression of QUAS-mCD8-GFP. (c) Control: GMR-QF2/+; alrm-Gal4/+; QUAS-mCD8GFP/+. AttA RNAi, GMR-QF2/+; alrm-Gal4/UAS-AttA RNA; QUAS-mCD8GFP/+. CecA RNAi, GMR-QF2/+; alrm-Gal4/+; UAS-CecA RNAi/QUAS-mCD8GFP. (PDF 289 kb) [file 12974_2018_1278_MOESM6_ESM.pdf]

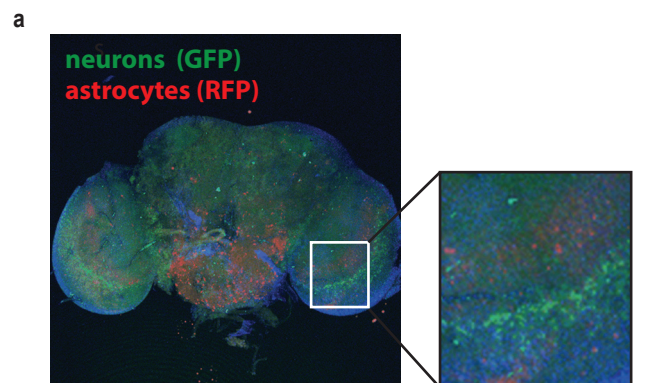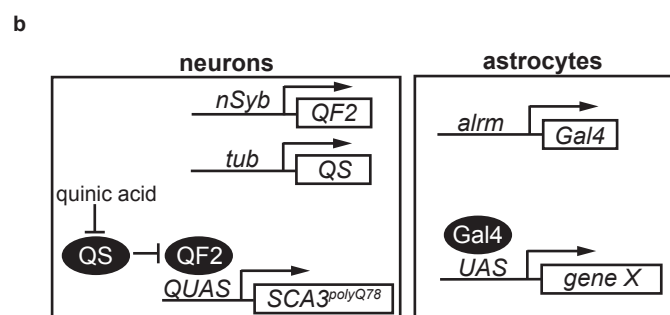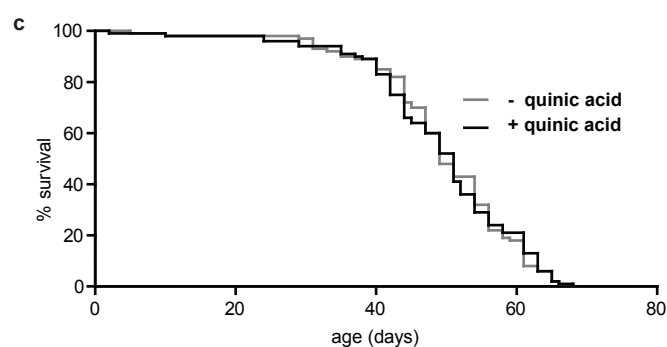

**d** Relish knockdown at 18°C

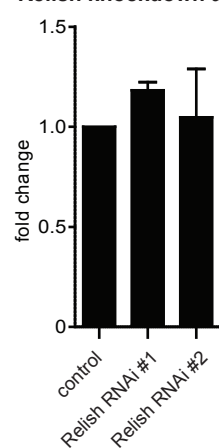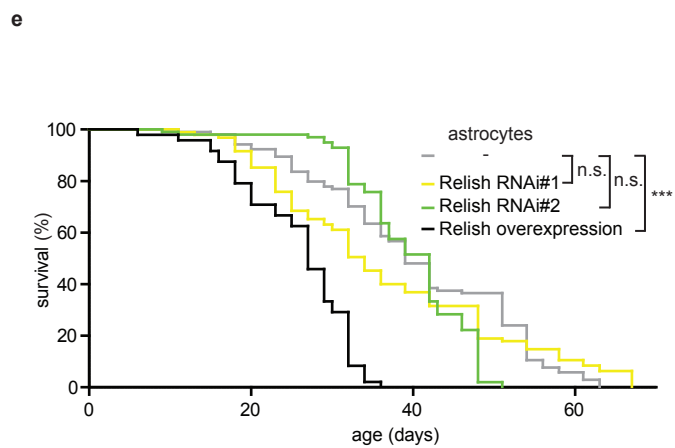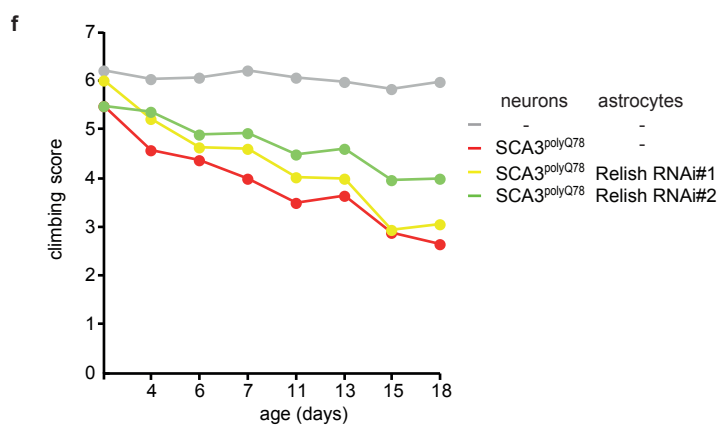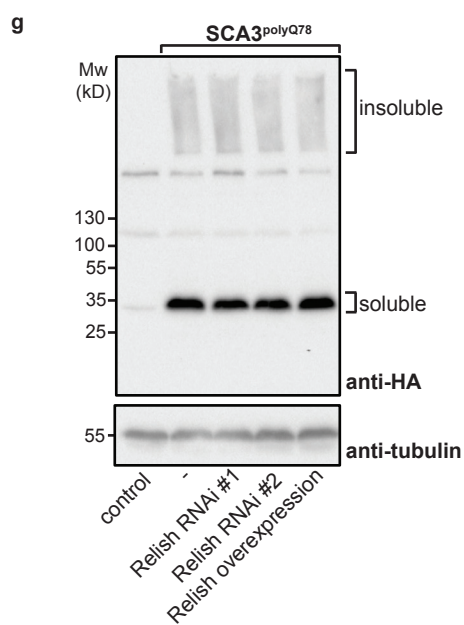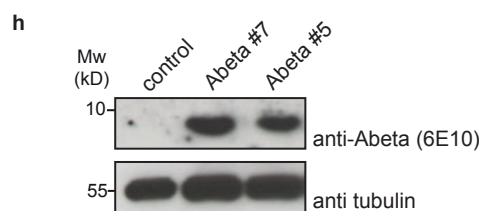

Supplement: Supplementary file 7 — a The UAS-Gal4 and the QUAS-QF system function independently. QUAS-QF-driven GFP expression specifically in neurons (GFP); UAS-Gal4-driven expression of myr-RFP specifically in astrocytes (RFP). Nuclei are stained with Hoechst (blue). b Genetic setup to inducibly express SCA3polyQ78 in neurons and simultaneously modulate gene expression in astrocytes. QUAS-QF was used to express SCA3polyQ78; UAS-Gal4 was used to modulate expression in astrocytes. Neuronally expressed QF2 (expressed under control of the pan-neuronal nSyb promoter) is suppressed by QS (expressed under control of the tubulin (tub) promoter). This suppression is alleviated by quinic acid, resulting in expression of SCA3polyQ78. For details on the fly lines used, see experimental procedures. c Quinic acid does not affect lifespan. Control flies were cultured on standard fly food with or without quinic acid, and the fraction of dead flies was determined over time. d Raising flies at 18 °C does not induce expression of Relish RNAi constructs. Progeny of flies ubiquitously expressing RNAi constructs targeting Relish (Relish RNAi #1 and Relish RNAi #2) raised at 18 °C were analyzed for expression of Relish as in Additional file 4b. e Effect of modulating expression of Relish in astrocytes on lifespan. The lifespan of control flies or flies expressing Relish RNAi or overexpression constructs specifically in astrocytes was analyzed. f Effect of modulating Relish expression in astrocytes on impairment of mobility induced by neuronal SCA3polyQ78 expression. Control flies or flies expressing inducible SCA3polyQ78 together with Relish RNAi or Relish overexpression constructs targeted to astrocytes were analyzed for mobility over time. Scoring was done as described in the experimental procedures. g Effect of modulating Relish expression in astrocytes on SCA3polyQ78 levels or aggregation. Head lysates of 15-day-old flies as in Fig. 5c were analyzed for expression of HA-tagged SCA3polyQ78 on western blot. Tubulin was [file 12974_2018_1278_MOESM7_ESM.pdf]
