# Supplementary material for: Inhibition of NF-κB in astrocytes is sufficient to delay neurodegeneration induced by proteotoxicity in neurons
Source: J Neuroinflammation. 2018 Sep 11;15:261. doi: 10.1186/s12974-018-1278-2 (PMC6134576; doi:10.1186/s12974-018-1278-2)
Supplement: Supplementary file 3 — RNAi screen to determine the contribution of genes in astrocytes to SCA3polyQ78-induced eye degeneration. Flies expressing SCA3polyQ78 in the eyes together with alrm-Gal4 were crossed to fly lines containing UAS-RNAi constructs to specifically knock down genes in astrocytes. Progeny expressing SCA3polyQ78 in the eyes with individual genes in astrocytes knocked down were analyzed for SCA3polyQ78-induced eye degeneration. The extent of degeneration was quantified and plotted in a table. The percentage of degeneration in SCA3polyQ78 eyes was set at 100% and the effect of RNAi-mediated gene knockdown in astrocytes was determined by the percentage of deviation from the control. The suppressors of degeneration (in which RNAi-induced downregulation of gene expression in astrocytes enhanced the SCA3polyQ78 phenotype, 60% or more deviation from the control) are shown in orange, the enhancers (40% or more deviation from the control) in purple. Genotype of the crosses SCA3polyQ78, GMR-QF2/+; QUAS-SCA3polyQ78:: alrm-Gal4/+. RNAi: GMR-QF2/+; QUAS-SCA3polyQ78:: alrm-Gal4/+ together with UAS-RNAi. (PDF 149 kb) [file 12974_2018_1278_MOESM3_ESM.pdf]

Signaling		
gene		VDRC stock number
CG17336, Lcch3	212,48%	109606-KK
CG6378, SPARC	168,48%	100566-KK
CG8394, VGAT	141,03%	103586-KK
CG18176, defl	117,46%	20604-GD
CG16827, ItgaPS4	109,47%	109783-KK
CG3143, foxo	103,78%	107786-KK
CG3408	101,19%	36306-GD
CG3022, GABA-B-R3	91,30%	108036-KK
CG6357	82,35%	8782-GD
CG10997, Clic	81,09%	105975-KK
CG3173, IntS1	74,74%	25825-GD
CG5195, atk	74,74%	100110-KK
CG5372, ItgaPS5	73,21%	100120-KK
CG10233, rtp	76,06%	109000-KK
CG4845, psidin	73,67%	103558-KK
CG4641,nwk	71,63%	102133-KK
CG17262, cnir	70,19%	104009-KK
CG8639, Cirl	63,66%	100749-KK
CG8909	62,50%	108629-KK
CG8250, Alk	58,89%	107083-KK
CG7449, hbs	55,33%	105913-KK
CG1411, CRMP	55,33%	101510-KK
CG11335, lox	53,95%	107435-KK
CG4604, Glaz	52,65%	107433-KK
CG42611, mgl	50,00%	105071-KK
CG31221	48,37%	103017-KK
CG15274, GABA-B-R1	47,15%	105863-KK
CG34399, Nox	46,27%	100753-KK
CG9453, Spn42Da	44,36%	106306-KK
CG18870	40,09%	100135-KK
CG11303, TM4SF	36,76%	8847-GD
CG17800, Dscam1	33,73%	108835-KK
CG7446, Grd	33,14%	5329-GD
CG2736	32,95%	102672-KK
CG6692, Cp1	32,62%	110619-KK
CG8434, lbk	32,44%	106679-KK
CG31094, LpR1	32,41%	106364-KK
CG13984	29,91%	101831-KK
CG42613	29,26%	102823-KK
CG11217, CanB2	25,08%	104370-KK
CG3131, Duox	24,36%	2593-GD
CG10537, Rdl	24,26%	100429-KK
CG33126, Nlaz	24,26%	101321-KK
CG34370	22,97%	100162-KK
CG6072, Sra	22,75%	107573-KK
CG4280, crq	22,54%	45883-GD
CG34385, dpr12	21,57%	44741-GD
CG33950, trol	20,00%	110494-KK
CG7586, Mcr	19,17%	100197 KK
CG4636, SCAR	18,93%	21908-GD
CG4167, Hsp67Ba	18,74%	104341-KK
CG7000, snmp1	17,00%	104210-KK
CG4096	16,04%	109025-KK
CG4168	13,91%	100080-KK
CG7228, pes	9,55%	100391-KK
CG31094, LpR1	8,73%	106364-KK
CG4821, teq	8,33%	15362-GD
CG31619, nolo	6,12%	104736-KK
CG6817, foi	6,05%	10102-GD
CG1804, Kek6	2,52%	109681-KK
CG12079, ND-30	1,67%	103412-KK
CG18525, Spn88Ea	-1,81%	28340-GD
CG14162, dpr6	-5,38%	103521-KK
CG12004	-5,52%	101732-KK
CG33087, LRP1	-10,78%	109605-KK
CG4545, SerT	-11,71%	11346-GD
CG30361, mtt	-12,63%	102982-KK
CG9623, if	-13,39%	100770-KK
CG1618, comt	-13,80%	105552-KK
CG8942, NimC1	-15,18%	105799-KK
CG10590, TM9SF3	-21,85%	110679-KK
CG7052, Tep2	-23,67%	106997-KK
CG1632	-24,42%	106107-KK
CG8743, Trpml	-25,44%	108088-KK
CG11372, galectin	-30,71%	107054-KK
CG1358	-34,35%	101453-KK
CG7509	-40,55%	51584-GD
CG30040, jeb	-43,48%	103047-KK
CG8329	-45,83%	101603-KK
CG18525, Spn88Ea	-45,83%	28340-GD
CG31092, LpR2	-48,30%	107597-KK
CG1771, mew	-50,51%	109608-KK
CG2086, drpr	-51,37%	4833-GD
CG6706, GABA-B-R2	-63,53%	1785-GD
CG42610, Fhos	-63,53%	34035-GD
CG7563, CalpA	-67,89%	101294-KK
CG1732, Gat		106638-KK
CG6378, SPARC		16678-GD
CG4099, Sr-CI		110014-KK
CG8095, scb		100949-KK

Immunity		
gene		VDRC stock number
CG7121, Tehao	80,14%	109705-KK
CG5528, Toll-9	66,80%	109635-KK
CG9681, PGRP-SB1	58,00%	101298-KK
CG43119, Ect4	54,79%	102044-KK
CG7250, Toll-6	46,77%	27103-GD
CG7250, Toll-6	42,69%	928-GD
CG6531, wgn	41,70%	9152-GD
CG5490, Tl	38,64%	100078-KK
CG6794, Dif	25,97%	100537-KK
CG4432, PGRP-LC	12,80%	101633-KK
CG6667, dl	7,23%	45996-GD
CG1857, nec	0,17%	108366-KK
CG4437, PGRP-LF	-3,68%	108313-KK
CG3048, Traf4	-4,02%	110766-KK
CG12919, egr	-8,27%	108814-KK
CG13422, GNBP-like3	-8,82%	107358-KK
CG5008, GNBP3	-17,65%	37256-GD
CG12489, dnr1	-20,49%	106453-KK
CG33717, PGRP-LD	-21,00%	51023-GD
CG7486, Dredd	-23,20%	104726-KK
CG6134, spz	-24,69%	105017-KK
CG11992, Rel	-30,75%	49414-GD
CG14746, PGRP-SC1a	-33,00%	43201-GD
CG6890, Tollo	-33,90%	27099-GD
CG43119, Ect4	-44,57%	105369-KK
CG14928, SPZ-4	-45,35%	7679-GD
CG8896, 18w	-46,49%	963-GD
CG6890, Tollo	-49,84%	9431-GD
CG8995, PGRP-LE	-51,14%	108199-KK
CG11992, Rel	-56,70%	49413-GD
CG11709, PGRP-SA	-57,00%	5594-GD

Neuropeptides and Neuropeptide receptors		
gene		VDRC stock number
CG14358, CCHa1	156,06%	104974-KK
CG13758, Pdfr	85,75%	106381-KK
CG7665, Lgr1	77,92%	104877-KK
CG14375, CCHa2	73,21%	102257-KK
CG14734, Tk	63,59%	103662-KK
CG8784, PK2-R1	52,80%	103822-KK
CG7105, Proc	52,00%	102488-KK
CG10342, NPF	49,00%	108772-KK
CG2872, AstA-R1	46,70%	101395-KK
CG10698, CrzR	45,90%	108506-KK
CG13480, LK	44,00%	14091-GD
CG6438, amon	36,00%	110788-KK
CG32540, CCKLR-17D3	32,05%	102039 KK
CG6456, Mip	31,00%	106076-KK
CG5811, Rya-R	26,80%	103973-KK
CG7395, sNPF-R	25,90%	9379-GD
CG1147, NPFR	25,40%	9605-GD
CG30340	24,10%	100088-KK
CG13633, AstA	20,50%	103215-KK
CG13061, Nplp3	16,00%	105584-KK
CG40733, RYa	15,48%	109264-KK
CG7887, TkR99D	15,48%	43329-GD
CG3302, Crz	14,10%	102204-KK
CG3441, Nplp1	13,00%	14035-GD
CG14593, CCHa2-R	10,74%	100290-KK
CG7285, AstC-R1	1,30%	110739-KK
CG13419, Burs	-1,00%	111063-KK
CG10823, SIFaR	-11,80%	1783-GD
CG14575, CapaR	-12,41%	105556-KK
CG7285, AstC-R1	-14,41%	13560-GD
CG9918, PK1-R	-20,75%	101115-KK
CG8795, PK2-R2	-23,10%	100927-KK
CG6440, Ms	-27,00%	108760-KK
CG15520, capa	-28,00%	41124-GD
CG14919, AstC	-34,00%	102735-KK
CG6515,TkR86C	-37,64%	107090-KK
CG33696, CNMaR	-40,00%	101076-KK
CG30106, CCHa1-R	-40,56%	103055-KK
CG11051, Nplp2	-54,00%	15305-GD

220%	suppressors
180%	
120%	
60%	
20%	
0%	
-20%	
-40%	enhancers
-100%	
	lethal

Li_Dijkers Additional File 3
