# Supplementary material for: Inhibition of NF-κB in astrocytes is sufficient to delay neurodegeneration induced by proteotoxicity in neurons
Source: J Neuroinflammation. 2018 Sep 11;15:261. doi: 10.1186/s12974-018-1278-2 (PMC6134576; doi:10.1186/s12974-018-1278-2)
Supplement: Supplementary file 4 — a Activation of Relish in SCA3polyQ78-expressing eyes predominantly occurred in astrocytes. (top) Representative picture of a control eye expressing GFP under the control of the Attacin promoter (att-GFP) and astrocytes expressing RFP or an eye coexpressing SCA3polyQ78. Pictures were taken from live eyes. (bottom) Representative picture of a dissected eye expressing SCA3polyQ78, together with att-GFP and RFP-expressing astrocytes. Nuclei are stained with Hoechst (blue). Note that in the bottom panel there is only an overlap between the RFP and GFP signal in the ommatidia. This may cause the imperfect overlap between the RFP and GFP signal in SCA3polyQ78-expressing flies in the top panel: the RFP signal from the underlying astrocytes. b Efficacy of Relish knockdown. Flies expressing daughterless-Gal4 (da-Gal4, ubiquitously expressed) were crossed to control flies (w1118) or fly lines containing RNAi constructs targeting Relish (Relish RNAi #1 and Relish RNAi #2) and expression of Relish in the progeny was determined. c Modulation of expression in astrocytes does not affect morphology of control eyes. Control flies or fly lines containing RNAi constructs targeting Relish (Relish RNAi #1 and Relish RNAi #2) or expressing Relish were crossed to alrm-Gal4 flies, and the morphology of the eyes was determined. d No cell-autonomous effect of Relish on the degenerative SCA3polyQ78 eye phenotype. Constructs targeting Relish were coexpressed in the eyes expressing SCA3polyQ78. e Expression of Relish RNAi in astrocytes does not influence the relocation of the astrocytes to the eye induced by eye-specific expression of SCA3polyQ78. Images of multiple flies to show similar levels of RFP in the eyes between flies expressing eye-specific of SCA3polyQ78 and astrocyte-specific myr-RFP (RFP) in the absence or presence of Relish RNAi targeted to astrocytes. f Modulating Relish expression in astrocytes does not affect levels of mCD8-GFP in the eyes. Lysates of fly heads expressing eye-s [file 12974_2018_1278_MOESM4_ESM.pdf]

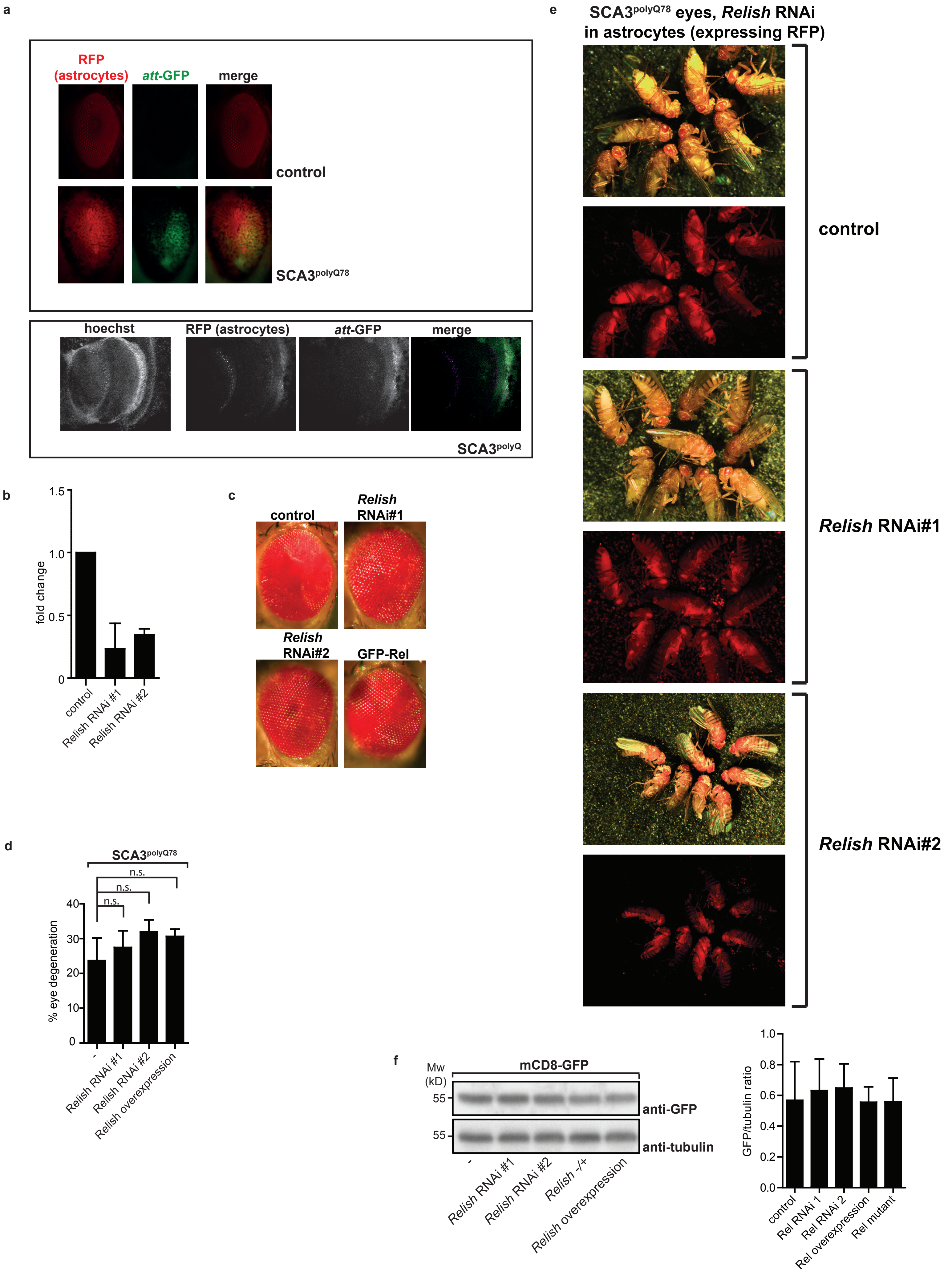
a
RFP (astrocytes)
att-GFP
merge
control
SCA3polyQ78
hoechst
RFP (astrocytes)
att-GFP
merge
SCA3polyQ
b
fold change
1.5
1.0
0.5
0
control
Relish RNAi #1
Relish RNAi #2
c
control
Relish RNAi#1
Relish RNAi#2
GFP-Rel
d
SCA3polyQ78
n.s.
n.s.
n.s.
% eye degeneration
40
30
20
10
0
-
Relish RNAi #1
Relish RNAi #2
Relish overexpression
e
SCA3polyQ78 eyes, Relish RNAi in astrocytes (expressing RFP)
control
Relish RNAi#1
Relish RNAi#2
f
Mw (kD)
mCD8-GFP
55
55
anti-GFP
anti-tubulin
-
Relish RNAi #1
Relish RNAi #2
Relish -/+
Relish overexpression
GFP/tubulin ratio
1.0
0.8
0.6
0.4
0.2
0.0
control
Rel RNAi 1
Rel RNAi 2
Rel overexpression
Rel mutant
